# Reliable Diagnostic Tests and Thresholds for Preoperative Diagnosis of Non‐Inflammatory Arthritis Periprosthetic Joint Infection: A Meta‐analysis and Systematic Review

**DOI:** 10.1111/os.13500

**Published:** 2022-10-01

**Authors:** Haozheng Tang, Jialian Xu, Wei'en Yuan, You Wang, Bing Yue, Xinhua Qu

**Affiliations:** ^1^ Department of Bone and Joint Surgery, Department of Orthopedics Renji Hospital, Shanghai Jiao Tong University School of Medicine Shanghai China; ^2^ Ministry of Education Engineering Research Center of Cell & Therapeutic Antibody School of Pharmacy, Shanghai Jiao Tong University Shanghai China

**Keywords:** Diagnosis, Meta‐analysis, Periprosthetic joint infection, Serum and synovial test, Threshold effect, Total joint arthroplasty

## Abstract

**Objective:**

The current diagnostic criteria for periprosthetic joint infection (PJI) are diverse and controversial, leading to delayed diagnosis. This study aimed to evaluate and unify their diagnostic accuracy and the threshold selection of serum and synovial routine tests for PJI at an early stage.

**Methods:**

We searched the MEDLINE and Embase databases for retrospective or prospective studies which reported preoperative‐available assays (serum, synovial, or culture tests) for the diagnosis of chronic PJI among inflammatory arthritis (IA) or non‐IA populations from January 1, 2000 to June 30, 2022. Threshold effective analysis was performed on synovial polymorphonuclear neutrophils (PMN%), synovial white blood cell (WBC), serum C‐reactive protein (CRP), and erythrocyte sedimentation rate (ESR) to find the relevant cut‐offs.

**Results:**

Two hundred and sixteen studies and information from 45,316 individuals were included in the final analysis. Synovial laboratory‐based α‐defensin and calprotectin had the best comprehensive sensitivity (0.91 [0.86–0.94], 0.95 [0.88–0.98]) and specificity (0.96 [0.94‐0.97], 0.95 [0.89–0.98]) values. According to the threshold effect analysis, the recommended cut‐offs are 70% (sensitivity 0.89 [0.85–0.92], specificity 0.90 [0.87–0.93]), 4100/μL (sensitivity 0.90 [0.87–0.93], specificity 0.97 [0.93–0.98]), 13.5 mg/L (sensitivity 0.84 [0.78–0.89], specificity 0.83 [0.73–0.89]), and 30 mm/h (sensitivity 0.79 [0.74–0.83], specificity 0.78 [0.72–0.83]) for synovial PMN%, synovial WBC, serum CRP, and ESR, respectively, and tests seem to be more reliable among non‐IA patients.

**Conclusions:**

The laboratory‐based synovial α‐defensin and synovial calprotectin are the two best independent preoperative diagnostic tests for PJI. A cut off of 70% for synovial PMN% and tighter cut‐offs for synovial WBC and serum CRP could have a better diagnostic accuracy for non‐IA patients with chronic PJI.

## Graphical abstract

This work provides additional insights and brand‐new evidences to the diagnostic accuracy of traditional and novel tests in early diagnosis of periprosthetic joint infection (PJI), especially the threshold selection. The results could attract more attentions on finding reliable cut‐offs and further influence the update of present criteria, which might provide significant meanings to the diagnosis and therapy strategy of PJI.

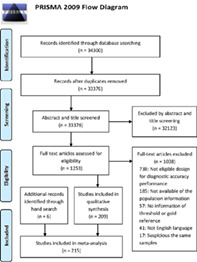

## Introduction

Different guidelines, criteria, and articles recommend diverse tests for periprosthetic joint infection (PJI), and the research on this subject is continuously updated, creating further confusion.^1^, ^2^ Positive cultures of tissues or joint fluids extracted from patients are the most intuitionistic evidence supporting direct PJI diagnosis, but the high rates of delayed or missed diagnosis is the main constraint. The Musculoskeletal Infection Society (MSIS) criteria,^3^, ^4^ one of the most globally accepted PJI diagnostic strategies, selects aspiration culture and the presence of the sinus tract as the major criteria and chooses other routine serum and synovial tests like serum C‐reactive protein (CRP), erythrocyte sedimentation rate (ESR), synovial white blood cell (WBC), and synovial polymorphonuclear neutrophils (PMN%) as minor criteria, similar to the diagnostic criteria published by the Infectious Diseases Society of America^5^ and the European Bone and Joint Infection Society criteria.^6^ Although the MSIS criteria present a reliable definition of PJI, it does not aid diagnosis at the very early stage of disease when infection occurs, because the tissue and aspiration culture require days or even weeks for the pathogen to grow.

The tests that can be performed before revision surgery have also been discussed widely, their various cut‐offs, especially the routine tests like serum CRP and synovial WBC, showed controversial diagnostic performance in different studies. In addition, the different gold standards selected in various articles may also lead to the over‐ or under‐estimation of the preoperative diagnostic accuracy of histological markers.^1^, ^2^, ^7^, ^8^ Furthermore, patients with inflammatory arthritis (IA), including inflammatory autoimmune arthritis, such as rheumatoid arthritis, who are undergoing immunosuppressive treatment have not only a higher risk of PJI, but the level of their inflammatory markers, especially synovial WBC and PMN%, have also been reported to be extremely similar with those of ordinary PJI patients, further complicating PJI diagnosis.^9^, ^10^ Moreover, the most important facts are probably interfering with the diagnostic accuracy of these tests, and the cut‐offs selected before are based mainly on experts' consensus, without scientific evidence or evaluation.^11^ Several experts have previously noticed this problem.^12^ After the 2018 International Consensus Meeting (ICM), the updated consensus on PJI has added new tests and altered the cut‐offs of PMN% from 80% to 70%, which verified that the cut‐offs selection had received enough attention.^13^, ^14^ Herein, a systematic review by Carli *et al*.^1^ tried to unify these ambiguities, but reported only a composite conclusion that synovial tests have a better holistic diagnostic capability than serum‐ or tissue‐based tests, maintaining the study with excess IA population and ignoring the mistiness of thresholds. Additionally, their work did not refer the results to a unified gold standard like the MSIS criteria.

The primary objective of this study was to identify the independent tests and their reliable cut‐offs for the diagnosis of chronic PJI with satisfactory diagnostic capability. The secondary objective was to separately evaluate the diagnostic performance of the tests among non‐IA and IA populations.

## Methods and Materials

### Search Strategy and Selection Criteria

This meta‐analysis and systematic review was performed according to the Cochrane Collaboration guidelines.^15^ Prospective and retrospective studies were included if they provided information on the diagnostic test accuracy of one or more preoperative biomarkers for PJI diagnosis. We included studies published from January 2000 to June 2022 by systematically searching MEDLINE and EMBASE databases, using a search string based on MeSH (Appendix S1). Inclusion criteria were: (i) studies with data on specific information of preoperative accessible biomarkers from patients who needed revision surgery for the treatment of PJI after knee or hip joint arthroplasty; (ii) studies with sufficient data to calculate the number of true‐positive (TP), false‐negative (FN), false‐positive (FP), and true‐negative (TN) patients with and without IA in the study; (iii) studies that reported a clear “gold standard”; and (iv) the cutoffs reported. Exclusion criteria were: (i) studies demonstrated the invalid information including patients with shoulder or other arthroplasties; (ii) studies reported the diagnostic biomarkers accessible only from revision surgery progress, such as periprosthetic tissue or sonicate fluid for culture; (iii) studies without extractive data for diagnostic test accuracy, such as TP, FP, FN, and TN; (iv) studies that provided equivocal gold standard or reference diagnostic methods; (v) research included suspicious same group of the patients; (vi) cadaver studies; and (vii) studies not in English.

### Data Extraction

All independently extracted articles were screened and reviewed independently by two researchers (TH and YH), and any controversy was resolved by a third reviewer (QX). One investigator (TH) extracted the data, including patient characteristics and biomarker details. To maximize the number of the studies and retain more calculable information, we retained all studies which reported the partial information mentioned above, and recorded the details for further subgroup analysis. The suspected duplicated data was deleted and retained only once in the end.

### Data Analysis and Threshold Effective Analysis

Statistical analyses were performed using the Stata/SE version 17.1 (Stata Corp, College Station, TX, USA).^16^ R software (The R Project, Vienna, Austria) was used for additional statistical analysis. Two reviewers (TH and JX) calculated the TP, FP, FN, and TN using relevant data extracted from included studies to independently evaluate the subsequent diagnostic accuracy of PJI.^17^ The forest plot analysis, positive and negative likelihood ratios, diagnostic odds ratio (DOR), and the area under the curve of the summary receiver operating characteristic curves (AUCs) were analyzed among different diagnostic markers.^18^, ^19^ Fagan's nomograms were used to demonstrate the post‐test probability, and likelihood ratio scattergrams were used to evaluate the clinical application value of different markers. Publication bias was estimated using the Deek's funnel plot asymmetry test.^20^

The threshold level was determined by trial and error, including the selection of turning points along a predefined interval, after which the turning point that gave the maximum model likelihood was chosen. We conducted a log likelihood ratio test comparing the one‐line linear regression model with the two‐piecewise linear model.^21^ Heterogeneity calculation was conducted using Cochran's Q‐test. The random effects model was used to evaluate the diagnostic accuracy when the result showed significant heterogeneity (*I* ^2^ > 50%), while the fixed effects model was used when the result showed minor heterogeneity (*I* ^2^ < 50%).^22^ We divided the original sample into different subgroups based on the IA comorbidities, types of arthroplasty, and gold standards for heterogeneity analysis.^18^ We used the Quality Assessment of Diagnostic Accuracy Studies 2 (QUADAS‐2) tool to evaluate the quality of the articles.^23^

## Results

### Study Selection

**Fig. 1:**
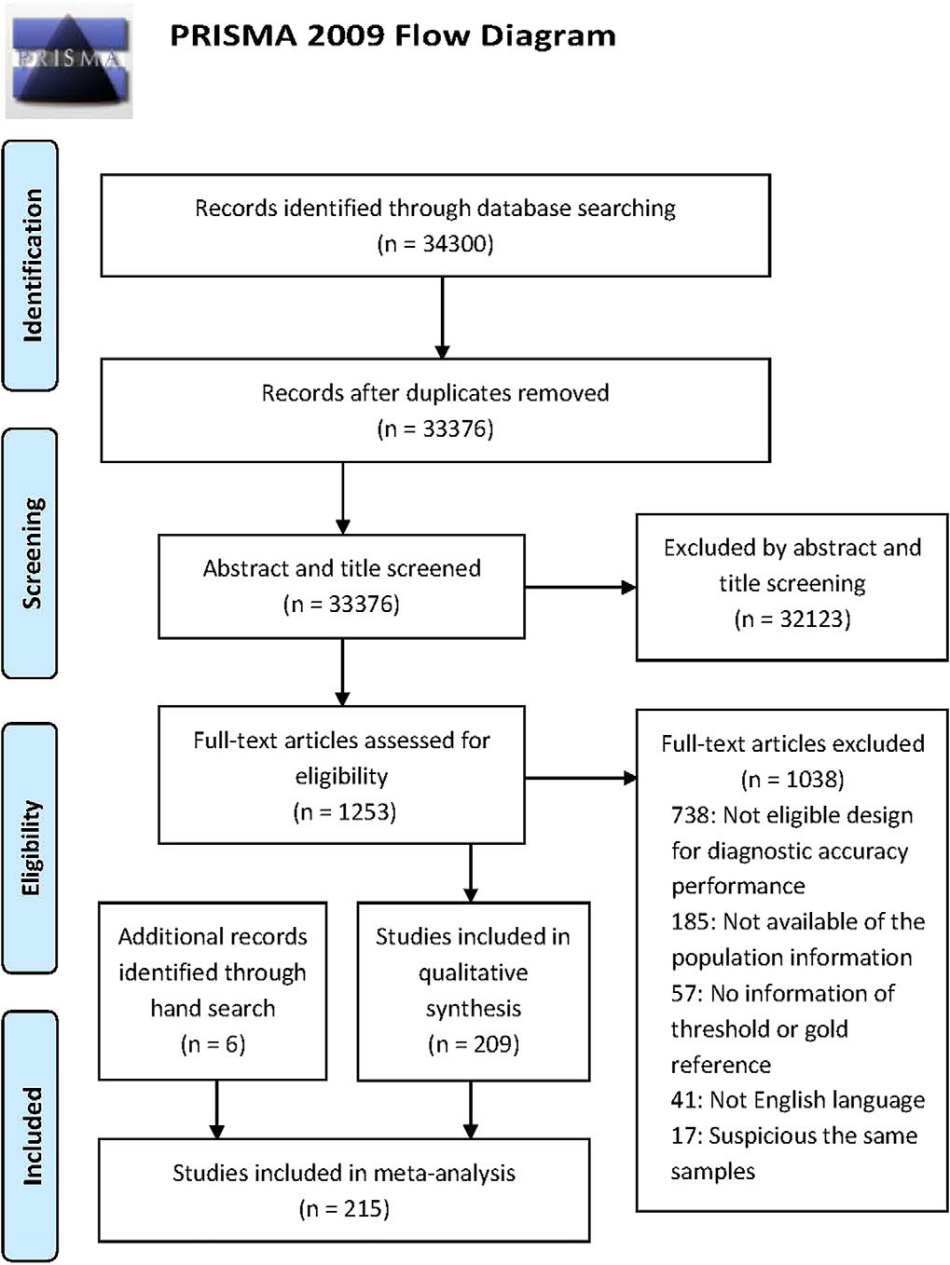
Flow diagram of the included studies

Final analysis was performed on 215 articles with six additional studies searched manually, as shown in Fig. Fig. 1, and the QUADAS‐2 score criteria revealed that most of the extracted studies had high risks. Excluding brand new markers that only appeared recently and have limited data, 129 records reported diagnostic information of serum markers (serum CRP, ESR, procalcitonin, IL‐6, D‐Dimer, WBC, platelet count [PLT], neutrophil to lymphocyte ratio [NLR], monocyte to lymphocyte ratio [MLR], platelet to lymphocyte ratio [PLR], platelet to mean platelet volume ratio [PVR], FDP [fibrin degradation product] and Fibrinogen),^9^, ^10^, ^11^, ^24^, ^25^, ^26^, ^27^, ^28^, ^29^, ^30^, ^31^, ^32^, ^33^, ^34^, ^35^, ^36^, ^37^, ^38^, ^39^, ^40^, ^41^, ^42^, ^43^, ^44^, ^45^, ^46^, ^47^, ^48^, ^49^, ^50^, ^51^, ^52^, ^53^, ^54^, ^55^, ^56^, ^57^, ^58^, ^59^, ^60^, ^61^, ^62^, ^63^, ^64^, ^65^, ^66^, ^67^, ^68^, ^69^, ^70^, ^71^, ^72^, ^73^, ^74^, ^75^, ^76^, ^77^, ^78^, ^79^, ^80^, ^81^, ^82^, ^83^, ^84^, ^85^, ^86^, ^87^, ^88^, ^89^, ^90^, ^91^, ^92^, ^93^, ^94^, ^95^, ^96^, ^97^, ^98^, ^99^, ^100^, ^101^, ^102^, ^103^, ^104^, ^105^, ^106^, ^107^, ^108^, ^109^, ^110^, ^111^, ^112^, ^113^, ^114^, ^115^, ^116^, ^117^, ^118^, ^119^, ^120^, ^121^, ^122^, ^123^, ^124^, ^125^, ^126^, ^127^, ^128^, ^129^, ^130^, ^131^, ^132^, ^133^, ^134^, ^135^, ^136^, ^137^, ^138^, ^139^, ^140^, ^141^, ^142^, ^143^, ^144^, ^145^, ^146^, ^147^, ^148^, ^149^ 114 studies reported synovial markers (synovial WBC, PMN%, IL‐6, IL‐1β, CRP, TNF‐α, Calprotectin, and leucocyte esterase [LE] and laboratory or lateral‐flow based α‐defensin test),^10^, ^29^, ^31^, ^34^, ^36^, ^43^, ^49^, ^51^, ^52^, ^58^, ^59^, ^61^, ^62^, ^64^, ^65^, ^72^, ^73^, ^74^, ^76^, ^77^, ^79^, ^80^, ^81^, ^84^, ^85^, ^86^, ^87^, ^88^, ^90^, ^95^, ^97^, ^99^, ^117^, ^118^, ^122^, ^124^, ^125^, ^127^, ^129^, ^132^, ^135^, ^136^, ^137^, ^138^, ^142^, ^144^, ^150^, ^151^, ^152^, ^153^, ^154^, ^155^, ^156^, ^157^, ^158^, ^159^, ^160^, ^161^, ^162^, ^163^, ^164^, ^165^, ^166^, ^167^, ^168^, ^169^, ^170^, ^171^, ^172^, ^173^, ^174^, ^175^, ^176^, ^177^, ^178^, ^179^, ^180^, ^181^, ^182^, ^183^, ^184^, ^185^, ^186^, ^187^, ^188^, ^189^, ^190^, ^191^, ^192^, ^193^, ^194^, ^195^, ^196^, ^197^, ^198^, ^199^, ^200^, ^201^, ^202^, ^203^, ^204^, ^205^, ^206^, ^207^, ^208^, ^209^, ^210^, ^211^, ^212^, ^213^, ^214^, ^215^, ^216^, ^217^ and 36 studies reported aspiration culture.^24^, ^25^, ^26^, ^31^, ^35^, ^36^, ^40^, ^44^, ^62^, ^81^, ^82^, ^83^, ^84^, ^96^, ^97^, ^115^, ^130^, ^138^, ^197^, ^198^, ^209^, ^210^, ^218^, ^219^, ^220^, ^221^, ^222^, ^223^, ^224^, ^225^, ^226^, ^227^, ^228^, ^229^, ^230^, ^231^, ^232^, ^233^ for chronic PJI (Appendix S2). All 24 biomarkers tests were evaluated on the basis of ≥2 articles, with only synovial TNF‐α evaluated on the basis of only two articles.

### Main Characteristics

All synovial biomarkers but TNF‐α showed AUC ≥ 0.9. The diagnostic accuracy of most synovial tests performed better than serum tests and aspiration culture, while laboratory‐based α‐defensin test (ELISA) and calprotectin test are the two best among the 10 synovial tests in the chronic PJI population. Among all 13 serum tests, serum IL‐6 has the highest DOR and AUC. We then maintained only the studies containing IA patients lower than 5% (chronic PJI without IA population) for subsequent analysis, achieving similar results (Table 1 and Figs [Link], [Link]).

**TABLE 1:** Diagnostic test accuracy of serum, synovial tests and aspiration culture

	Studies Number	SEN (95% CI)	SPE (95% CI)	AUC (95% CI)	Positive‐LR (95% CI)	Negative‐LR (95% CI)	DOR (95% CI)	*I* ^2^ (*P* value)
*Chronic PJI*								
Serum tests								
CRP	120	0.82 (0.80, 0.84)	0.79 (0.76, 0.81)	0.87 (0.84, 0.90)	3.9 (3.5, 4.4)	0.23 (0.20, 0.26)	17 (14, 21)	100% (*P* < 0.001)
ESR	92	0.79 (0.76, 0.82)	0.78 (0.74, 0.81)	0.85 (0.82, 0.88)	3.5 (3.1, 4.0)	0.27 (0.24, 0.31)	13 (10, 16)	100% (*P* < 0.001)
IL‐6	19	0.86 (0.78, 0.92)	0.83 (0.76, 0.88)	0.91 (0.88, 0.93)	5.1 (3.6, 7.3)	0.17 (0.10, 0.27)	31 (16, 60)	99% (*P* < 0.001)
D‐dimer	24	0.77 (0.69, 0.83)	0.75 (0.66, 0.82)	0.82 (0.79, 0.85)	3.0 (2.3, 4.0)	0.31 (0.24, 0.41)	10 (6, 15)	99% (*P* < 0.001)
PCT	12	0.57 (0.43, 0.70)	0.90 (0.79, 0.95)	0.81 (0.77, 0.84)	5.6 (2.9, 10.8)	0.48 (0.36, 0.63)	12 (6, 23)	99% (*P* < 0.001)
WBC	25	0.45 (0.35, 0.56)	0.86 (0.78, 0.92)	0.71 (0.67, 0.75)	3.3 (2.3, 4.8)	0.63 (0.54, 0.74)	5 (3, 8)	100% (*P* < 0.001)
PLT	5	0.63 (0.59, 0.67)	0.73 (0.67, 0.78)	0.70 (0.66, 0.74)	2.3 (1.9, 2.8)	0.51 (0.45, 0.57)	5 (4, 6)	70% (*P* = 0.019)
NLR	8	0.70 (0.65, 0.74)	0.74 (0.70, 0.77)	0.78 (0.74, 0.81)	2.7 (2.2, 3.2)	0.41 (0.34, 0.49)	6 (4, 9)	0% (*P* = 0.500)
MLR	4	0.69 (0.57, 0.79)	0.80 (0.77, 0.82)	0.80 (0.77, 0.84)	3.4 (2.8, 4.1)	0.39 (0.27, 0.56)	9 (5, 15)	91% (*P* < 0.001)
PLR	4	0.77 (0.73, 0.80)	0.72 (0.57, 0.83)	0.79 (0.75, 0.82)	2.7 (1.7, 4.3)	0.32 (0.25, 0.42)	8 (4, 17)	92% (*P* < 0.001)
PVR	10	0.71 (0.61, 0.80)	0.75 (0.70, 0.79)	0.79 (0.75, 0.82)	2.8 (2.4, 3.3)	0.38 (0.28, 0.53)	7 (5, 11)	99% (*P* < 0.001)
Fibrinogen	15	0.80 (0.74, 0.85)	0.84 (0.80, 0.87)	0.89 (0.86, 0.91)	4.9 (4.1, 5.8)	0.24 (0.19, 0.31)	20 (16, 26)	95% (*P* < 0.001)
FDP	7	0.73 (0.67, 0.78)	0.70 (0.63, 0.75)	0.77 (0.73, 0.81)	2.4 (1.9, 3.0)	0.39 (0.30, 0.51)	6 (4, 10)	0% (*P* = 0.464)
Synovial tests								
sWBC	50	0.87 (0.84, 0.90)	0. 90 (0.87, 0.92)	0.95 (0.92, 0.96)	8.7 (6.8, 11.2)	0.14 (0.11, 0.18)	62 (42, 92)	99% (*P* < 0.001)
PMN%	50	0.88 (0.85, 0.89)	0.88 (0.85, 0.90)	0.94 (0.91, 0.95)	7.1 (5.7, 8.9)	0.14 (0.12, 0.17)	50 (36, 69)	99% (*P* < 0.001)
sCRP	25	0.87 (0.84, 0.90)	0.93 (0.89, 0.95)	0.94 (0.91, 0.96)	12.0 (8.2, 17.7)	0.14 (0.11, 0.17)	85 (59, 123)	99% (*P* < 0.001)
sIL‐1b	7	0.88 (0.70, 0.96)	0.92 (0.85, 0.96)	0.95 (0.93, 0.97)	10.6 (5.2, 21.8)	0.13 (0.04, 0.38)	83 (15, 456)	23% (*P* = 0.136)
sIL‐6	11	0.82 (0.74, 0.88)	0.95 (0.87, 0.98)	0.93 (0.90, 0.95)	15.0 (6.2, 36.3)	0.19 (0.13, 0.28)	80 (27, 239)	96% (*P* < 0.001)
α‐defensin *Lab‐based*	16	0.91 (0.86, 0.94)	0.96 (0.94, 0.97)	0.98 (0.96, 0.99)	21.3 (14.4, 31.4)	0.10 (0.06, 0.14)	221 (114, 428)	88% (*P* = 0.211)
α‐defensin *Lateral Flow*	17	0.85 (0.78, 0.90)	0.96 (0.95, 0.97)	0.98 (0.96, 0.99)	22.5 (15.8, 31.9)	0.15 (0.10, 0.23)	146 (92, 232)	98% (*P* < 0.001)
LE	19	0.88 (0.81, 0.92)	0.94 (0.91, 0.96)	0.97 (0.95, 0.98)	14.0 (9.3, 20.9)	0.13 (0.09, 0.21)	105 (58, 189)	99% (*P* < 0.001)
sTNF‐α	4	0.76 (0.69, 0.82)	0.81 (0.69, 0.89)	0.80 (0.75, 0.84)	4.1 (2.4, 6.8)	0.29 (0.22, 0.38)	14 (7, 27)	86% (*P* < 0.001)
Calprotectin	6	0.95 (0.88, 0.98)	0.95 (0.89, 0.98)	0.99 (0.97, 0.99)	20.1 (7.9, 50.8)	0.05 (0.02, 0.14)	371 (70, 1959)	0% (*P* = 0.494)
Aspiration culture								
Joint fluid	36	0.65 (0.58, 0.72)	0.97 (0.95, 0.98)	0.92 (0.89, 0.94)	20.5 (12.8, 32.9)	0.36 (0.30, 0.44)	57 (33, 98)	98% (*P* < 0.001)
*Chronic PJI without IA Population*								
Serum tests								
CRP	106	0.81 (0.79, 0.84)	0.79 (0.76, 0.81)	0.87 (0.84, 0.90)	3.8 (3.4, 4.3)	0.24 (0.21, 0.27)	16 (13, 20)	100% (*P* < 0.001)
ESR	82	0.78 (0.75, 0.81)	0.78 (0.75, 0.81)	0.85 (0.82, 0.88)	3.6 (3.2, 4.1)	0.28 (0.24, 0.32)	13 (11, 16)	100% (*P* < 0.001)
IL‐6	16	0.84 (0.73, 0.92)	0.84 (0.78, 0.89)	0.91 (0.88, 0.93)	5.4 (3.8, 7.7)	0.19 (0.10, 0.34)	29 (13, 65)	98% (*P* < 0.001)
D‐dimer	23	0.76 (0.69, 0.82)	0.75 (0.66, 0.82)	0.82 (0.79, 0.85)	3.1 (2.3, 4.1)	0.32 (0.24, 0.41)	10 (6, 15)	99% (*P* < 0.001)
PCT	9	0.58 (0.40, 0.75)	0.84 (0.69, 0.93)	0.79 (0.75, 0.82)	3.7 (2.1, 6.6)	0.49 (0.35, 0.70)	8 (4, 14)	99% (*P* < 0.001)
WBC	23	0.45 (0.34, 0.57)	0.87 (0.79, 0.92)	0.72 (0.68, 0.76)	3.5 (2.3, 5.3)	0.63 (0.53, 0.74)	6 (3, 9)	100% (*P* < 0.001)
PLT	5	0.63 (0.59, 0.67)	0.73 (0.67, 0.78)	0.70 (0.66, 0.74)	2.3 (1.9, 2.8)	0.51 (0.45, 0.57)	5 (4, 6)	70% (*P* = 0.019)
NLR	8	0.70 (0.65, 0.74)	0.74 (0.70, 0.77)	0.78 (0.74, 0.81)	2.7 (2.2, 3.2)	0.41 (0.34, 0.49)	6 (4, 9)	0% (*P* = 0.500)
MLR	4	0.69 (0.57, 0.79)	0.80 (0.77, 0.82)	0.80 (0.77, 0.84)	3.4 (2.8, 4.1)	0.39 (0.27, 0.56)	9 (5, 15)	91% (*P* < 0.001)
PLR	4	0.77 (0.73, 0.80)	0.72 (0.57, 0.83)	0.79 (0.75, 0.82)	2.7 (1.7, 4.3)	0.32 (0.25, 0.42)	8 (4, 17)	92% (*P* < 0.001)
PVR	9	0.73 (0.62, 0.82)	0.74 (0.69, 0.78)	0.79 (0.75, 0.82)	2.8 (2.4, 3.3)	0.37 (0.26, 0.52)	8 (5, 12)	99% (*P* < 0.001)
Fibrinogen	14	0.80 (0.74, 0.85)	0.83 (0.79, 0.87)	0.89 (0.86, 0.91)	4.8 (4.0, 5.7)	0.24 (0.18, 0.31)	20 (16, 26)	95% (*P* < 0.001)
FDP	7	0.73 (0.67, 0.78)	0.70 (0.63, 0.75)	0.77 (0.73, 0.81)	2.4 (1.9, 3.0)	0.39 (0.30, 0.51)	6 (4, 10)	0% (*P* = 0.464)
Synovial tests								
sWBC	47	0.87 (0.83, 0.90)	0.90 (0.87, 0.92)	0.95 (0.92, 0.96)	8.7 (6.5, 11.4)	0.15 (0.11, 0.19)	59 (38, 93)	99% (*P* < 0.001)
PMN%	45	0.88 (0.85, 0.90)	0.89 (0.86, 0.92)	0.94 (0.91, 0.96)	8.0 (6.1, 10.3)	0.14 (0.12, 0.17)	58 (41, 82)	99% (*P* < 0.001)
sCRP	19	0.88 (0.85, 0.91)	0.94 (0.90, 0.96)	0.95 (0.93, 0.97)	14.6 (9.1, 23.2)	0.12 (0.09, 0.16)	118 (76, 182)	98% (*P* < 0.001)
sIL‐1b	4	0.91 (0.52, 0.99)	0.94 (0.82, 0.98)	0.97 (0.95, 0.98)	15.8 (4.2, 59.6)	0.09 (0.01, 0.77)	167 (7, 4271)	0% (*P* = 0.214)
sIL‐6	7	0.80 (0.63, 0.90)	0.95 (0.80, 0.99)	0.93 (0.91, 0.95)	17.4 (3.4, 88.9)	0.21 (0.10, 0.42)	83 (10, 674)	93% (*P* < 0.001)
α‐defensin *Lab‐based*	16	0.91 (0.88, 0.94)	0.96 (0.93, 0.97)	0.98 (0.96, 0.99)	20.7 (13.6, 31.4)	0.09 (0.06, 0.13)	230 (116, 454)	0% (*P* = 0.433)
α‐defensin *Lateral Flow*	17	0.85 (0.78, 0.90)	0.96 (0.95, 0.97)	0.98 (0.96, 0.99)	22.5 (15.8, 31.9)	0.15 (0.10, 0.23)	146 (92, 232)	98% (*P* < 0.001)
LE	22	0.88 (0.82, 0.93)	0.94 (0.90, 0.96)	0.97 (0.95, 0.98)	14.5 (9.3, 22.6)	0.12 (0.08, 0.20)	117 (63, 218)	99% (*P* < 0.001)
sTNF‐α	2	0.81 (0.70, 0.89)	0.72 (0.64, 0.78)	0.84 (0.78, 0.88)	2.9 (2.2, 3.8)	0.26 (0.16, 0.44)	11 (5, 22)	100% (*P* < 0.001)
Calprotectin	5	0.95 (0.85, 0.98)	0.95 (0.87, 0.98)	0.99 (0.97, 0.99)	20.3 (6.8, 60.7)	0.06 (0.02, 0.17)	364 (51, 2604)	0% (*P* = 0.492)
Aspiration culture								
Joint fluid	35	0.65 (0.58, 0.72)	0.97 (0.95, 0.98)	0.92 (0.89, 0.94)	19.7 (12.3, 31.5)	0.36 (0.30, 0.44)	55 (32, 94)	98% (***p*** < 0.001)

Abbreviations: AUC, area under the ROC curve; DOR, diagnostic odds ratio; FDP, fibrin degradation product; IA, inflammatory arthritis; LE, leucocyte esterase; LR, likelihood ratio; MLR, monocyte to lymphocyte ratio; NLR, neutrophil to lymphocyte ratio; PCT, serum procalcitonin; PLR, platelet to lymphocyte ratio; PLT, platelet count; PMN%, proportion of neutrophils in synovial fluid; PVR, platelet to mean platelet volume ratio; sCRP, synovial CRP; sIL‐6, synovial IL‐6; sIL‐1β, synovial IL‐1β; SEN, sensitivity; SPE, specificity; sTNF‐α, synovial TNF‐α; sWBC, synovial WBC.

### Subgroup and Threshold Analysis

Threshold effect regression analysis on four routine markers (serum CRP, ESR, synovial WBC, and synovial PMN%) for chronic PJI diagnosis among the non‐IA population revealed different results, with three having new cut‐offs: 13.5 mg/L, 4100/μL and 70% for serum CRP, synovial WBC and synovial PMN%, respectively (Table 2). We also conducted subgroup analysis and hierarchical analysis with groups by thresholds to verify the newly updated recommendation, and the results proved their validity (Appendices S3 and S4). However, ESR showed quasi‐linear performance during the analysis. The results after grouping the cut‐offs showed that the traditional cut‐off, 30 mm/h, appeared to have the best performance. In the subgroup with MSIS criteria as the gold standard, new cut‐offs were proved to have better diagnostic capability in some situations. (Appendix S5).

**TABLE 2:** Comparation between traditional cutoffs and new cutoffs based on threshold effect analysis (chronic PJI without IA population)

	Studies number	SEN (95% CI)	SPE (95% CI)	AUC (95% CI)	Positive‐LR (95% CI)	Negative‐LR (95% CI)	DOR (95% CI)	*I* ^2^ (*P* value)
PMN (%)								
80	16	0.84 (0.80, 0.88)	0.94 (0.90, 0.97)	0.93 (0.90, 0.95)	14.6 (8.4, 25.4)	0.17 (0.13, 0.21)	88 (50, 155)	98% (*P* < 0.001)
70 (69–73)	12	0.89 (0.85, 0.92)	0.90 (0.87, 0.93)	0.95 (0.93, 0.97)	9.1 (6.7, 12.3)	0.12 (0.09, 0.17)	75 (46, 120)	66% (*P* = 0.027)
sWBC (/μL)								
3000	19	0.88 (0.85, 0.90)	0.92 (0.88, 0.95)	0.94 (0.91, 0.96)	11.1 (7.2, 17.2)	0.13 (0.11, 0.17)	83 (47, 145)	97% (*P* < 0.001)
4100 *(3966–4450)*	3	0.90 (0.87, 0.93)	0.97 (0.93, 0.98)	0.91 (0.89, 0.94)	27.1 (13.3, 55.2)	0.10 (0.07, 0.13)	274 (121, 624)	86% (*P* < 0.001)
CRP (mg/L)								
10	56	0.81 (0.77, 0.85)	0.78 (0.74, 0.82)	0.87 (0.83, 0.89)	3.7 (3.2, 4.4)	0.24 (0.20, 0.29)	16 (12, 20)	100% (*P* < 0.001)
13.5 *(12.5–14.5)*	6	0.84 (0.78, 0.89)	0.83 (0.73, 0.89)	0.90 (0.87, 0.92)	4.8 (3.1, 7.6)	0.19 (0.13, 0.27)	26 (14, 46)	86% (*P* < 0.001)
ESR (mm/h)								
25–29	7	0.73 (0.65, 0.80)	0.82 (0.74, 0.88)	0.82 (0.79, 0.85)	4.2 (2.9, 6.0)	0.33 (0.26, 0.41)	13 (9, 9)	92% (*P* < 0.001)
30	47	0.79 (0.74, 0.83)	0.78 (0.72, 0.83)	0.85 (0.82, 0.88)	3.5 (2.8, 4.4)	0.27 (0.22, 0.33)	13 (10, 18)	100% (*P* < 0.001)
30–39	15	0.77 (0.69, 0.84)	0.78 (0.72, 0.83)	0.84 (0.81, 0.87)	3.5 (2.7, 4.5)	0.29 (0.21, 0.40)	12 (7, 20)	96% (*P* < 0.001)
40	3	0.56 (0.42, 0.68)	0.79 (0.65, 0.88)	0.71 (0.68, 0.77)	2.6 (1.6, 4.2)	0.56 (0.43, 0.75)	5 (2, 9)	91% (*P* < 0.001)
40–49	12	0.71 (0.64, 0.77)	0.79 (0.74, 0.84)	0.82 (0.79, 0.85)	3.4 (2.7, 4.4)	0.37 (0.29, 0.47)	9 (6, 15)	97% (*P* < 0.001)

Abbreviations: AUC, area under the ROC curve; DOR, diagnostic odds ratio; IA, inflammatory arthritis; LR, likelihood ratio; PMN%, proportion of neutrophils in synovial fluid; SEN, sensitivity; SPE, specificity; sWBC, synovial WBC.

Among the articles included, those with information regarding the independent IA population available were also isolated^9^, ^10^, ^49^, ^86^, ^133^, ^144^, ^203^ for the subgroup analysis. The diagnostic accuracy of the four routine tests in two groups revealed that all four markers had better diagnostic performance in non‐IA (Table 3). In addition, following the suggestion from previous reviews, we performed a subgroup analysis using the MSIS criteria as the gold standard. The performance of laboratory‐based α‐defensin test was corroborated by the significant diagnostic capability with MSIS as the gold standard (Table 4).

**TABLE 3:** Comparation between inflammatory and non‐inflammatory Arthritis

	Studies Number	SEN (95% CI)	SPE (95% CI)	AUC (95% CI)	Positive‐LR (95% CI)	Negative‐LR (95% CI)	DOR (95% CI)	*I* ^2^ (*P* value)
PMN%								
Non‐inflammatory	18	0.90 (0.87, 0.92)	0.91 (0.87, 0.94)	0.95 (0.93, 0.97)	10.2 (6.9, 15.0)	0.11 (0.09, 0.14)	92 (56, 152)	97% (*P* < 0.001)
Inflammatory	5	0.93 (0.82, 0.98)	0.85 (0.80, 0.89)	0.89 (0.86, 0.91)	6.1 (4.6, 8.2)	0.08 (0.03, 0.22)	74 (26, 214)	22% (*P* = 0.138)
sWBC								
Non‐inflammatory	14	0.89 (0.86, 0.91)	0.93 (0.89, 0.95)	0.94 (0.92, 0.96)	12.4 (8.2, 18.6)	0.12 (0.10, 0.15)	100 (61, 163)	96% (*P* < 0.001)
Inflammatory	4	0.90 (0.83, 0.94)	0.81 (0.75, 0.85)	0.92 (0.90, 0.94)	4.6 (3.5, 5.8)	0.12 (0.07, 0.21)	37 (19, 73)	100% (*P* = 0.500)
CRP								
Non‐inflammatory	48	0.84 (0.82, 0.87)	0.78 (0.74, 0.82)	0.89 (0.85, 0.91)	3.8 (3.2, 4.6)	0.20 (0.17, 0.24)	19 (14, 25)	100% (*P* < 0.001)
Inflammatory	6	0.84 (0.78, 0.90)	0.75 (0.46, 0.88)	0.87 (0.84, 0.89)	3.4 (1.4, 8.5)	0.21 (0.15, 0.28)	17 (6, 48)	96% (*P* < 0.001)
ESR								
Non‐inflammatory	38	0.81 (0.76, 0.84)	0.79 (0.76, 0.82)	0.86 (0.83, 0.89)	3.9 (3.4, 4.4)	0.24 (0.20, 0.30)	16 (12, 20)	99% (*P* < 0.001)
Inflammatory	6	0.80 (0.71, 0.87)	0.71 (0.56, 0.83)	0.83 (0.80, 0.86)	2.8 (1.6, 4.8)	0.28 (0.17, 0.47)	10 (4, 28)	55% (*P* = 0.054)

Abbreviations: AUC, area under the ROC curve; DOR, diagnostic odds ratio; LR, likelihood ratio; PMN%, proportion of neutrophils in synovial fluid; SEN, sensitivity; SPE, specificity; sWBC, synovial WBC

**TABLE 4:** Diagnostic test accuracy of the tests with MSIS criteria as gold standard

	Studies Number	SEN (95% CI)	SPE (95% CI)	AUC (95% CI)	Positive‐LR (95% CI)	Negative‐LR (95% CI)	DOR (95% CI)	*I* ^2^ (*P* value)
Serum test								
CRP	53	0.83 (0.80, 0.85)	0.78 (0.73, 0.81)	0.87 (0.84, 0.90)	3.7 (3.1, 4.4)	0.22 (0.20, 0.26)	17 (13, 22)	100% (*P* < 0.001)
ESR	47	0.77 (0.73, 0.81)	0.81 (0.78, 0.84)	0.86 (0.83, 0.89)	4.1 (3.6, 4.7)	0.28 (0.24, 0.33)	15 (12, 19)	99% (*P* < 0.001)
D‐dimer	20	0.75 (0.66, 0.81)	0.78 (0.69, 0.85)	0.83 (0.79, 0.86)	3.4 (2.4, 4.8)	0.33 (0.25, 0.44)	10 (6, 17)	99% (*P* < 0.001)
Synovial test								
sWBC	18	0.86 (0.83, 0.88)	0.90 (0.86, 0.94)	0.91 (0.89, 0.94)	8.9 (6.0, 13.3)	0.16 (0.13, 0.19)	56 (34, 93)	97% (*P* < 0.001)
PMN %	17	0.87 (0.84, 0.90)	0.87 (0.81, 0.91)	0.92 (0.89, 0.94)	6.5 (4.5, 9.4)	0.15 (0.12, 0.19)	43 (26, 71)	97% (*P* < 0.001)
LE	12	0.85 (0.76, 0.91)	0.94 (0.90, 0.96)	0.96 (0.94, 0.97)	13.6 (8.5, 22.0)	0.16 (0.10, 0.26)	85 (43, 170)	99% (*P* < 0.001)
α‐defensin *Lab‐test*	12	0.92 (0.86, 0.95)	0.96 (0.94, 0.98)	0.98 (0.97, 0, 99)	24.1 (15.3, 37.8)	0.09 (0.05, 0.14)	283 (129, 622)	33% (*P* = 0.113)
α‐defensin *Lateral flow*	16	0.89 (0.84, 0.93)	0.96 (0.94, 0.97)	0.98 (0.96, 0.99)	21.3 (13.9, 32.6)	0.11 (0.08, 0.17)	188 (110, 321)	97% (*P* < 0.001)
sCRP	15	0.88 (0.84, 0.91)	0.92 (0.86, 0.95)	0.94 (0.92, 0.96)	10.6 (6.2, 18.2)	0.13 (0.10, 0.18)	80 (49, 133)	99% (*P* < 0.001)
**Preoperative Culture**								
Aspiration culture	10	0.66 (0.50, 0.79)	0.97 (0.95, 0.98)	0.97 (0.95, 0.98)	22.5 (11.1, 45.7)	0.35 (0.23, 0.55)	64 (23, 178)	77% (*P* = 0.007)

Abbreviations: AUC, area under the ROC curve; DOR, diagnostic odds ratio; LE, leucocyte esterase; LR, likelihood ratio; PCT, serum procalcitonin; PMN%, proportion of neutrophils in synovial fluid; sIL‐6, synovial IL‐6; sTNF‐α, synovial TNF‐α; sIL‐1β, synovial IL‐1β; SEN, sensitivity; SPE, specificity; sWBC, synovial white blood cell; sCRP, synovial C‐reactive protein.

## Discussion

Our study not only systematic reviewed the biomarkers with preoperative diagnosis of PJI which were reported since 2000, but also reviewed cut‐off value of multiple commonly used clinical biomarkers such as CRP via curve fitting method for the first time. The results showed that the synovial fluid tests performed better in PJI diagnosis than serum tests and aspiration culture. And among the plethora of synovial fluid tests, the lab‐based synovial α‐defensin test is the most satisfying independent preoperative diagnostic test for PJI, especially in the non‐inflammatory group. Furthermore, the statistical analysis underpinning the threshold effect analysis of PJI diagnosis was based on a liner regression model. We analyzed the cut‐off values of four common tests and provided more satisfactory new thresholds of them. Among them, we reduced the threshold of PMN% to 70%, which is completely consistent with the adjustment in MSIS 2018.

Currently, an independent test to definitively diagnose PJI has not yet been discovered. The updated definition of PJI (2018 ICM criteria) has been broadly recognized as a reliable guideline for diagnosis of PJI. This combines multiple evidence‐based tests to rate and then decide whether infection has occurred. However, the major criteria, pathogen culture, takes a long time to yield a result, and is vulnerable to being contaminated during the traumatic operation. Furthermore, the sinus tract with evidence of communication to the joint or visualization of the prosthesis often appears only in the late stage of PJI.^13^ Consequently, it is challenging to diagnose chronic PJI in the early period of infection. For chronic PJI, an earlier diagnosis is strongly linked to the development of less sequelae. Therefore, the importance of developing an accurate and convenient mechanism of early diagnosis for chronic PJI is obvious, and our study aimed to achieve this.

### Accuracy of Diagnostic Tests

In this comprehensive and systematic study, based mainly on the threshold effect analysis for preoperatively accessible chronic PJI diagnostic tests, laboratory‐based α‐defensin test (ELISA) with a regular threshold of 5.2 mg/L (equal to a semiquantitative signal‐to‐cut = off [S/CO] ratio of 1.0)^163^ was identified as having the highest recommended evaluation among the 24 evaluated tests. Moreover, the results from continuous subgroup analysis with MSIS criteria as the unified gold standard proved this conclusion (Tables 1 and 4). The superior performance of laboratory‐based α‐defensin test corresponds well with previous randomized studies and systematic reviews.^1^, ^186^, ^234^ However, low penetration and high price limit further popularization of this test in many countries. In contrast, calprotectin, the new synovial biomarker has the advantages of price and availability. Calprotectin is an innate immune protein mainly originated from neutrophils and macrophages.^235^ It will be secreted to resist bacterial infection when the inflammatory response occurs. Calprotectin has the promising potential of being an independent diagnostic biomarker for diagnosing preoperative PJI with its excellent sensitivity (0.95) and specificity (0.95). However, the included six studies about calprotectin test have high heterogeneity within diagnostic methods (ELISA, lateral flow assay, immunoturbidimetric immunoassay) and cut‐off values.

### Cut‐off Values and Threshold Effect Analysis

Consequently, we conducted the threshold effect analysis to the four routine tests, serum CRP, ESR, synovial WBC, and synovial PMN%, as the most popular and convenient tests for early PJI diagnosis. This is thus the first systematic review to explore the diagnostic cut‐offs of PJI. The results revealed that the updated cut‐offs of serum CRP (13.5 mg/L), synovial WBC (4100/μL), and synovial PMN% (70.0%) have better diagnostic accuracy than traditional values among non‐IA population, while the cut‐off of ESR remains at 30 mm/h (Table 2). This result also partially supported the newly updated guideline from the consensus of 2018 ICM.^14^ Although the sensitivity and specificity of the routine tests with updated cut‐offs cannot compare favorably with novel tests such as the laboratory‐based α‐defensin and calprotectin tests, this discovery nevertheless showed that the thresholds of the existing tests for PJI diagnosis are far from perfect.

Researchers continuously attempt to find more convenient and reliable methods to diagnose PJI at an early stage, given the multiple disadvantages of the current mainstream diagnostic methods, such as the insufficient diagnostic accuracy or delayed definition, while our work has also proved that the traditional bacterial culture has an unacceptable high FN rate. When the pre‐test probability of the PJI is at 80% (high clinical suspicion), the post‐test probability of PJI, provided that the culture is negative, is still 57% (Figure S23). Comprehensive diagnostic strategies like the MSIS criteria have provided solutions to these problems by promoting the combined use of the valuable serum and synovial tests. The newly updated MSIS criteria, one of the most popular diagnostic strategies, has added some novel reliable tests like d‐dimer and α‐defensin and modified the cut‐off of synovial PMN to 70%.^14^ In spite of this, the status of joint aspiration or pathological tissue culture appears to have never been threatened in the clinics, which is most likely because of the lack of dependable and objective evidence supporting the regulation including both additional tests and updated threshold. Based on the results of this, we supposed that the novel tests with significantly higher diagnostic accuracy will provide help better, as the tighter serum CRP and WBC counting might also enhance the specificity and reduce the misdiagnosis rate, while the looser PMN% could increase the sensitivity of early diagnosis of PJI at a very early stage instead.

This evidence‐based analysis aimed to evaluate and identify reliable tests with satisfying thresholds to provide reliable early diagnosis for chronic PJI. The results confirmed that synovial tests have better holistic diagnostic accuracy than both the serum tests and aspiration culture, while laboratory‐based α‐defensin tests showed the best performance with or without the unified MSIS criteria as the gold standard. First, we strongly recommended using the synovial α‐defensin ELISA test as a reliable diagnostic test for chronic PJI after excluding the IA diagnosis; while other synovial tests (except synovial IL‐6 and TNF‐α) could also be treated as surrogates if the α‐defensin ELISA test is unavailable in some areas. Some studies have previously evaluated the point‐of‐care lateral‐flow testing technique, Synovasure™ (Zimmer Biomet, Warsaw, IN, USA), which could provide results of synovial α‐defensin tests in only 10 min; however, its lower diagnostic capability and higher price restrict its widespread use.^189^ Second, use of the α‐defensin ELISA test might also be interfered by low‐virulence organisms caused infection, further studies focusing on this project should be promoted as current studies are still limited.^199^ Third, the synovial calprotectin test requires more prospective randomized trials to determine its diagnostic ability and the best diagnostic method.

Under this circumstance, although the diagnostic performance of routine tests like serum CRP, ESR, synovial WBC counting or PMN% is unsatisfactory, they will not be replaced in the near future, due to their ease‐of‐use. Based on the threshold effect analysis in this study, we believe that the sensitivity/specificity of the synovial PMN, WBC, and serum CRP can be elevated from 0.84/0.94 to 0.89/0.90, 0.88/0.92 to 0.90/0.97, and 0.81/0.78 to 0.84/0.83, respectively, while the threshold of ESR remains at 30 mm/h (Table 2). Especially, the results of synovial PMN first supported the revision that the cut‐off of PMN was changed from 80% to 70% in the 2018 MSIS criteria. Verification of the importance of the biomarkers' threshold is a significant finding. Although the other two new cut‐offs reported in this work still require verifications from further studies, but they purposed those tighter cut‐offs of serum CRP and synovial WBC counting could show better diagnostic performance. Further, updated recommendation of the thresholds was based mainly on the non‐IA population, and has also been confirmed by the hierarchical analysis (Appendices S3 and S4). With improved cut‐off values, the clinical practice of PJI diagnosis will be benefited by the threshold effect. Like looser cut‐offs hint more potential patients. In contrast, tighter cut‐offs will make diagnosis more accurate. Compared to the non‐IA group, the accuracy and sensitivity of four routine diagnostic tests for the IA patients are very low. There are several limitations that may limit the availability of diagnostic tests, including fluctuations of inflammatory factors and the influence of undergoing drug treatment for IA. Therefore, evaluating the cut‐off values for threshold effect is currently difficult among IA patients.

### Efforts to Reliable Diagnostic Tests for IA Patients

In clinical practice, the discrimination between PJI and the flare state of IA by histological tests is another challenge, as several inflammatory indicators of patients with IA in their acute flare stage will also be considerably increased. Additionally, immunosuppressive agents may increase the risk of infection and block the production of several inflammatory markers (serum CRP, IL‐6, etc.), which could impair accurate diagnosis of PJI. By ignoring this key problem, prior diagnostic guidelines and systematic reviews provided few recommendations; therefore, we also tried to evaluate the PJI diagnostic performance of four routine tests among patients with IA (Table 3). We found that the diagnostic accuracy of routine tests for chronic PJI was generally lower in the IA population. We think that the study included an excessive IA population, which could interfere with the interpretation of the systematic analysis of the diagnostic accuracy of the biomarkers; thus, future studies on the diagnosis of the PJI should exclude IA populations. Studies on PJI diagnosis targeting IA patients should also be conducted. Unfortunately, we were unable to provide more information due to the limited number of publications in this work. More information on this point is needed, and we believe that the ambiguity will be clarified in the near future.

### Strengths and Limitations

This study has several strengths. First, this is not only the most comprehensive and systematic evaluation of preoperative diagnostic biomarkers for PJI in this field, with the largest number of articles included, but is also the first to review cut‐off value of multiple commonly used clinical biomarkers, such as CRP, using the curve fitting method. Meanwhile, our work explored the diagnostic cut‐offs of PJI in a systematic review for the first time. Overall, we concluded that the cut‐offs selected previously for the majority of traditional testing biomarkers were inappropriate, which may be the ultimate cause of the continued inefficiency and confusing diagnostic efficacy of PJI diagnosis. In addition, with the method of threshold effect analysis, we confirmed the rationalization of the PMN% threshold revision in the 2018 MSIS. We found that lowering of the threshold for PMN from 80% to 70% increased the sensitivity/specificity and AUC of the synovial PMN from 0.84/0.94 to 0.89/0.90, 0.93 to 0.95. The widespread acceptance of the ICM criteria have been attributed to its universal popularity for PJI diagnosis. Nevertheless, this criteria has an unavoidable disadvantage for such criteria, in that the threshold of every test has to be renewed in conjunction with the most recent research. Our research showed that some criteria from the newly updated guidelines from the consensus of 2018 ICM have room for improvement. It is a significant finding to verify the importance of the biomarkers' threshold. We also provided a subgroup analysis of the studies using the MSIS criteria as the only gold standard. The results are also the same with and supporting the former conclusion. The activity period of IA frequently along with enhancement of inflammatory factors and effects from anti‐inflammatory treatment. Therefore, we divided patients into the IA and non‐IA groups to compare the discrepancies in commonly used biomarkers between these two kinds of patients.

Nevertheless, this study has some limitations which should be mentioned. First, this work only contained studies that reported data from chronic PJI‐suspicious patients who underwent total knee arthroplasty or total hip arthroplasty; hence, all conclusions from this study cannot be applied to the diagnosis of acute PJI and patients with shoulder or elbow arthroplasty. Second, many details or further analysis cannot be collected and conducted, such as whether or not IA patients were in active flare or the threshold effective analysis in non‐IA patients, due to the meta‐analysis design of this study. Further research providing patient‐based data or large samples of individuals based on a registration database on PJI diagnosis should be performed to verify our results. Third, there are many other potential candidates, such as serum toll‐like receptor 2,^236^ synovial d‐lactate,^237^ synovial lipocalin 2,^164^ and high‐sensitivity CRP,^63^ that may provide independent diagnostic capability of PJI diagnosis before revision surgery but require further investigation. Fourth, this study includes 215 articles, which would unavoidably introduce heterogeneity, as indicated by the high I^2^ value shown in Table 1. To combat this, we tried to minimize diagnosis bias by performing subgroup analysis. Further, although the risk of diagnosis bias cannot be completely avoided, statistically significant evidence of publication bias was not found in this study. Further, as the only study used the newly updated 2018 MSIS criteria as the good standard,^202^ further investigations should take special note of studies that use the new guidelines.

## Conclusion

According to the results of this study, synovial fluid tests have better diagnostic accuracy for PJI than serum indicators and aspiration culture, and the laboratory‐based α‐defensin has the potential to be an independent chronic PJI diagnostic biomarker among non‐IA population when a diagnostic cut‐off of 5.2 mg/L (1 S/CO) is selected. This was confirmed when compared with the results of the analysis that used the MSIS criteria as the single gold standard. The synovial calprotectin test also has outstanding diagnostic accuracy and the advantage of low cost compared with other tests. The best cut‐off value and diagnostic tool of it need further research to determine.

Overall, we suggest using newly updated thresholds for synovial PMN% (70%), as well as tighter suggested cut‐offs of synovial WBC (4100/μL), serum CRP (13.5 mg/L), which could improve the diagnostic performance of these four routine tests. We believe that this study could contribute to the preoperative diagnosis of PJI and provide relevant insights for future diagnostic strategies for PJI.

## Author Contributions

All authors had full access to the data in the study and take responsibility for the integrity of the data and the accuracy of the data analysis. Conceptualization: HT and JX. Methodology: WY and YW. Validation: XQ and BY. Investigation: BY. Formal Analysis: HT and JX. Formal Analysis: WY, YW, BY and XQ. Supervision: HT and QX. Project Administration: WY and YW. Funding Acquisition: BY.

## Supporting information


**Appendix S1** Systematic review search strategy

**Appendix S2** Information on the 215 studies included

**Appendix S3** Subgroup analysis of chronic PJI without IA population

**Appendix S4** Hierarchical analysis of thresholds (chronic PJI without IA population)

**Appendix S5** Hierarchical analysis of thresholds with MSIS criteria as gold standard (chronic PJI without IA population)

**Fig. S1** Alpha‐defensin Laboratory test

**Fig. S2** Alpha‐defensin Lateral Flow

**Fig. S3** Calprotectin

**Fig. S4** LE

**Fig. S5** Synovial WBC

**Fig. S6** Synovial PMN%

**Fig. S7** Synovial CRP

**Fig. S8** Synovial IL‐6

**Fig. S9** Synovial IL‐1β

**Fig. S10** Serum CRP

**Fig. S11** Serum ESR

**Fig. S12** Serum IL‐6

**Fig. S13** Serum D‐Dimer

**Fig. S14** Serum PCT

**Fig. S15** Serum WBC

**Fig. S16** Serum PLT

**Fig. S17** Serum NLR

**Fig. S18** Serum MLR

**Fig. S19** Serum PLR

**Fig. S20** Serum PVR

**Fig. S21** Serum Fibrinogen

**Fig. S22** Serum FDP

**Fig. S23** Aspiration Culture
