# Supplementary material for: Reliable Diagnostic Tests and Thresholds for Preoperative Diagnosis of Non‐Inflammatory Arthritis Periprosthetic Joint Infection: A Meta‐analysis and Systematic Review
Source: Orthop Surg. 2022 Oct 1;14(11):2822–36. doi: 10.1111/os.13500 (PMC9627080; doi:10.1111/os.13500)
Supplement: Supplementary file 1 — Appendix S1 Systematic review search strategy [file OS-14-2822-s023.pdf]

Appendix S1: Systematic review search strategy.

Database	Step	Terms	Results*
Pubmed	1	((((((((((“Infections” [Mesh]) OR “Arthroplasty” [Mesh]) Periprosthetic Joint Infection[Title/Abstract]) OR Prosthesis Related Infections[Title/Abstract]) OR Joint Replacement Infection[Title/Abstract]) OR infection[Title/Abstract]) OR septic arthritis[Title/Abstract]) OR knee arthroplasty[Title/Abstract]) OR hip arthroplasty[Title/Abstract])	1338473
	2	((((((((((((“Diagnosis” [Mesh]) OR “Sensitivity and Specificity” [Mesh]) OR "ROC Curve"[Mesh]) OR diagnostic accuracy[Title/Abstract]) OR diagnostic test[Title/Abstract]) OR diagnostic procedure[Title/Abstract]) OR receiver operating characteristic[Title/Abstract]) OR ROC[Title/Abstract]) OR true positive[Title/Abstract]) OR true negative[Title/Abstract]) OR false positive[Title/Abstract]) OR false negative[Title/Abstract])	9424249
	3	((((((“Serum” [Mesh]) OR “Synovial fluid” [Mesh]) OR “Clinical Laboratory Techniques” [Mesh]) OR fine needle aspiration [Title/Abstract]) OR aspiration culture[Title/Abstract]) OR culture[Title/Abstract])	3295482
	4	"2000/01/01"[PDat]: "2022/06/30"[PDat]	
	5	English [lang]	
	6	Human [Mesh]	
	7	#1 AND #2 AND #3 AND #4 AND #5 AND #6	**140684**
Embase	1	('periprosthetic joint infection'/exp OR 'periprosthetic joint infection' OR 'infection'/exp OR 'infection' OR 'replacement arthroplasty'/exp OR 'replacement arthroplasty' OR 'prosthesis related infections.ab.' OR 'joint replacement infection.ab.' OR 'knee arthroplasty.ab.' OR 'hip arthroplasty.ab.' OR 'septic arthritis.ab.') AND [embase]/lim	3607492
	2	('diagnosis'/exp OR 'diagnostic accuracy'/exp OR 'diagnostic error'/exp OR 'sensitivity and specificity'/exp OR 'receiver operating characteristic'/exp OR 'roc.ab.' OR 'diagnostic test.ab.' OR 'true positive.ab.' OR 'true negative.ab.' OR 'false positive.ab.' OR 'false negative.ab.') AND [embase]/lim	6663869
	3	('serum'/exp OR 'synovial fluid'/exp OR 'joint biopsy'/exp OR 'Laboratory test.ab.' OR 'biological marker'/exp OR 'aspiration culture.ab.' OR 'culture.ab.') AND [embase]/lim	539667

4	[1-1-2000]/sd NOT [30-06-2022]/sd	
5	[english]/lim	
6	[human]/lim	
7	(#1 AND #2 AND #3 AND #4 AND #5 AND #6)	**28962**

* Search date: 2022-07-08
