# Supplementary material for: Reliable Diagnostic Tests and Thresholds for Preoperative Diagnosis of Non‐Inflammatory Arthritis Periprosthetic Joint Infection: A Meta‐analysis and Systematic Review
Source: Orthop Surg. 2022 Oct 1;14(11):2822–36. doi: 10.1111/os.13500 (PMC9627080; doi:10.1111/os.13500)
Supplement: Supplementary file 2 — Appendix S2 Information on the 215 studies included [file OS-14-2822-s027.pdf]

Appendix S2: Information on the 215 studies included.

Study	Year	Country	Sample Number	Test	Joints	Reference Standard	Inflammatory Arthritis: Included IA Patents	Inflammatory Arthritis: Patients Number	QUADAS-2 Overall Judgement: Risk of Bias	QUADAS-2 Overall Judgement: Applicability
Teller et al.	2000	USA	137	ESR/Aspiration Culture	Knee/Hip	Intraoperative Culture	N/A[b]	N/A[c]	High	Low
Itasaka et al.	2001	Japan	48	CRP/ESR/WBC/Aspiration Culture	Hip	Compositive Standard	No	0	Unclear	Low
Somme et al.	2003	USA	107	Aspiration Culture	Hip	Intraoperative Culture	Yes	N/A	High	Low
Mason et al.	2003	USA	86	sWBC/PMN	Knee	Intraoperative Culture	Yes	4	High	High
Bernard et al.	2004	France	228	CRP/ESR/WBC/Aspiration Culture	Knee/Hip	Intraoperative Culture	N/A	N/A	Unclear	Unclear
Malhotra et al.	2004	Australia	41	Aspiration Culture	Hip	Intraoperative Culture	Yes	N/A	High	High
Williams et al.	2004	UK	273	Aspiration Culture	Hip	Intraoperative Culture	N/A	N/A	Low	Unclear
Trampuz et al.	2004	USA	133	sWBC/PMN	Knee	Compositive Standard	No	0	High	Unclear
Panousis et al.	2005	UK	92	CRP/ESR/Aspiration Culture	Knee/Hip	Compositive Standard	N/A	N/A	High	Unclear
Ali et al.	2005	UK	73	Aspiration Culture	Hip	Intraoperative Culture	N/A	N/A	Unclear	Unclear
Di Cesare et al.	2005	Finland	58	CRP/ESR/IL-6/WBC	Knee/Hip	Intraoperative Culture	No	0	Unclear	Unclear
Van den bekerom et al.	2006	Belgium	68	Aspiration Culture	Knee	Compositive Standard	N/A	N/A	High	Unclear
Parvizi et al.	2006	USA	168	sWBC/PMN	Knee/Hip	Compositive Standard	No	0	High	Unclear
Baré et al.	2006	Canada	242	CRP/ESR	Knee	Compositive Standard	N/A	N/A	High	Unclear
Della Valle et al.	2007	USA	94	CRP/ESR/sWBC/PMN/Aspiration Culture	Knee	Compositive Standard	N/A	N/A	High	Unclear
Trampuz et al.	2007	USA	140	Aspiration Culture	Knee/Hip	Compositive Standard	N/A	N/A	High	Low
Nilsdotter-Augustinsson et al.	2007	Sweden	124	CRP/ESR/sWBC/TNF-a/sIL-1β/sIL6	Hip	Compositive Standard	Yes	5	High	High
Bottner et al.	2007	Germany	78	CRP/ESR/IL-6/WBC/PCT	Knee/Hip	Intraoperative Culture	Yes	5	Unclear	Unclear

Greidanus et al.	2007	Canada	151	CRP/ESR	Knee	Intraoperative Culture	No	0	High	Low
Simonsen et al.	2007	Denmark	76	CRP/ESR/WBC	Hip	Compositive Standard	N/A	N/A	High	Unclear
Austin et al.	2008	USA	296	CRP/ESR/IL-6/sWBC/PMN/Aspiration Culture	Knee	Compositive Standard	No	0	High	High
Müller et al.	2008	Germany	50	CRP/WBC/Aspiration Culture	Hip	Compositive Standard	N/A	N/A	High	Unclear
Gallo et al.	2008	Czech	94	Aspiration Culture	Knee/Hip	Compositive Standard	N/A	N/A	High	Unclear
Schinsky et al.	2008	USA	201	CRP/ESR/sWBC/PMN	Hip	Compositive Standard	No	0	High	Unclear
Ghanem et al.	2008	USA	128	sWBC	Knee	Compositive Standard	No	0	High	Low
Ghanem et al.	2008	USA	429	sWBC/PMN	Knee	Compositive Standard	No	0	High	Low
Fink et al.	2008	Germany	145	CRP	Knee	Intraoperative Culture	Yes	16	Unclear	Unclear
Tohtz et al.	2009	Germany	64	CRP/ESR/WBC/Aspiration Culture	Hip	Compositive Standard	N/A	N/A	High	Unclear
Chevillotte et al.	2009	USA	204	CRP/ESR/WBC	Hip	Intraoperative Culture	Yes	29	High	Unclear
Ghanem et al.	2009	USA	479	CRP/ESR	Hip	Compositive Standard	No	0	High	Unclear
Morgan et al.	2009	USA	903	CRP/ESR/WBC	Knee	Compositive Standard	N/A	N/A	High	Unclear
Meermans et al.	2010	UK	120	Aspiration Culture	Knee/Hip	Intraoperative Culture	N/A	N/A	Unclear	Unclear
Deirmengian et al.	2010	USA	51	CRP/ESR/sWBC/PMN/sIL-1β	Knee/Hip	Intraoperative Culture	Yes	N/A	High	High
Buttaro et al.	2010	Italy	69	CRP/ESR/IL-6	Hip	Intraoperative Culture	No	0	High	Unclear
Piper et al.	2010	USA	518	CRP/ESR	Knee/Hip	Compositive Standard	No	0	High	Unclear
Worthington et al.	2010	UK	46	CRP/ESR/IL-6	Hip	Compositive Standard	N/A	N/A	High	Low
Parvizi et al.	2011	USA	59	CRP/sCRP	Knee	Compositive Standard	N/A	N/A	High	Unclear
Parvizi et al.	2011	USA	108	LE	Knee	Compositive Standard	Yes	1	High	Unclear
Jacovides et al.	2011	USA	74	sCRP/sIL-6	Knee/Hip	Compositive Standard	N/A	N/A	High	Unclear
Johnson et al.	2011	USA	113	CRP/ESR	Knee	Compositive Standard	N/A	N/A	High	Unclear
Randau et al.	2011	Germany	103	CRP/WBC/IL-6/PCT	Knee/Hip	Compositive Standard	N/A	N/A	High	Unclear
Cipriano et al.	2012	USA	871	CRP/ESR/sWBC/PMN	Knee/Hip	Compositive Standard	Yes	61	High	Low

Schwartz et al.	2012	Germany	172	CRP/ESR/sWBC/PMN	Knee	Compositive Standard	Yes	6	High	Low
Parvizi et al.	2012	USA	63	sCRP	Knee/Hip	Compositive Standard	Yes	11	High	Unclear
Mihalic et al.	2012	Slovenia	206	sWBC/PMN	Hip	Compositive Standard	N/A	N/A	High	Unclear
Wetters et al.	2012	USA	93	LE	Knee/Hip	Compositive Standard	N/A	N/A	High	Unclear
Zmistowski et al.	2012	USA	150	sWBC/PMN	Knee/Hip	Compositive Standard	N/A	N/A	High	Unclear
Fink et al.	2012	Germany	100	CRP	Hip	Compositive Standard	Yes	7	Unclear	High
Toossi et al.	2012	USA	1856	WBC	Knee/Hip	Compositive Standard	N/A	N/A	High	Low
Gollwitzer et al.	2013	Germany	35	IL-6/sTNF-a/sIL-6/sIL-1β	Knee/Hip	Compositive Standard	Yes	N/A	High	High
Wyles et al.	2013	USA	39	CRP/ESR/sWBC/PMN	Hip	Intraoperative Culture	N/A	N/A	High	Unclear
Dinneen et al.	2013	UK	75	sWBC/PMN	Knee/Hip	Compositive Standard	No	0	High	Unclear
Vanderstappen et al.	2013	Belgium	44	sCRP	Knee	Compositive Standard	N/A	N/A	High	Unclear
Alijanipour et al.	2013	USA	1962	CRP/ESR	Knee/Hip	MSIS	No	0	High	Unclear
Miyamae et al.	2013	Japan	81	CRP	Knee/Hip	Compositive Standard	No	0	High	Low
El-Khier et al.	2013	Egypt	40	IL-6	Knee/Hip	Intraoperative Culture	N/A	N/A	High	Low
Glehr et al.	2013	Austria	124	CRP/WBC/IL-6/PCT	Knee/Hip	Compositive Standard	N/A	N/A	High	Unclear
Bingham et al.	2014	USA	61	CRP/ESR/WBC/α-Defensin/Aspiration Culture	Knee/Hip	MSIS	No	0	High	High
Cross et al.	2014	USA	110	Aspiration Culture	Hip	Intraoperative Culture	N/A	N/A	High	Unclear
Ryu et al.	2014	Korea	89	Aspiration Culture	Knee	Compositive Standard	N/A	N/A	High	Unclear
Ronde-Oustau et al.	2014	France	31	CRP/sCRP	Knee	MSIS	Yes	2	High	Unclear
Tetreault et al.	2014	USA	119	CRP/sCRP	Knee/Hip	MSIS	Yes	11	High	Unclear
Randau et al.	2014	Germany	120	CRP/WBC/IL-6/PCT/sIL-6	Knee/Hip	Compositive Standard	No	0	High	Unclear
Deirmengian et al.	2014	USA	149	sCRP/α-Defensin	Knee/Hip	MSIS	Yes	N/A	High	High
Deirmengian et al.	2014	USA	95	sCRP/sIL-1β/sIL-6/α-Defensin	Knee/Hip	MSIS	Yes	11	High	Unclear
Tischler et al.	2014	USA	189	LE	Knee/Hip	MSIS	N/A	N/A	Unclear	Unclear

Chalmers et al.	2014	USA	253	sWBC/PMN	Hip	Compositive Standard	N/A	N/A	High	Unclear
Liu et al.	2014	USA	38	CRP/ESR	Knee	MSIS	Yes	8	High	Unclear
Milone et al.	2014	USA	98	CRP/ESR/WBC	Hip	Intraoperative Culture	No	0	High	High
Elgeidi et al.	2014	Egypt	44	CRP/ESR/IL-6/WBC	Knee/Hip	Compositive Standard	No	0	High	Unclear
Friedrich et al.	2014	Germany	120	CRP/WBC	Knee/Hip	Compositive Standard	No	0	High	Unclear
Cansü et al.	2014	Turkey	31	CRP/ESR	Hip	Intraoperative Culture	N/A	N/A	High	Unclear
Claassen et al.	2014	Germany	77	CRP/WBC	Knee	Compositive Standard	N/A	N/A	High	Unclear
Wu et al.	2014	China	156	CRP/ESR	Knee/Hip	Compositive Standard	N/A	N/A	High	Low
Shen et al.	2015	China	110	Aspiration Culture	Knee/Hip	Compositive Standard	No	0	High	Unclear
Omar et al.	2015	Germany	80	CRP/ESR/sWBC/PMN/sCRP	Hip	Compositive Standard	N/A	N/A	High	High
Buttaro et al.	2015	Argentina	76	sCRP	Hip	MSIS	No	0	High	Unclear
Shafafy	2015	UK	103	LE	Knee/Hip	IDSA	No	0	High	Unclear
Chalmers et al.	2015	USA	433	sWBC/PMN	Hip	Compositive Standard	N/A	N/A	High	Unclear
Yuan et al.	2015	China	71	CRP/WBC/PCT	Hip	Compositive Standard	No	0	High	Low
Hoell et al.	2016	Germany	115	CRP/sWBC	Knee/Hip	Intraoperative Culture	Yes	5	High	Low
Frangiamore et al.	2016	USA	78	CRP/ESR/α-Defensin	Knee/Hip	MSIS	N/A	N/A	High	Unclear
Kwon et al.	2016	USA	62	CRP/ESR/sWBC/PMN	Hip	MSIS	N/A	N/A	High	Unclear
Shah et al.	2016	USA	121	CRP/ESR/sWBC/PMN	Knee/Hip	MSIS	N/A	N/A	High	Unclear
Frangiamore et al.	2016	USA	90	sTNF-a/sIL-6/sIL-1β	Knee/Hip	MSIS	Yes	6	High	Unclear
Choi et al.	2016	USA	138	sWBC	Hip	MSIS	No	0	High	Unclear
Boettner et al.	2016	USA	77	sWBC	Knee/Hip	Intraoperative Culture	No	0	Unclear	Unclear
Kasparek et al.	2016	USA	40	α-Defensin	Knee/Hip	MSIS	N/A	N/A	High	Unclear
De Vecchi et al.	2016	Italy	129	sCRP/LE	Knee/Hip	Compositive Standard	N/A	N/A	High	Low
Claassen et al.	2016	Germany	32	CRP/WBC	Knee	Compositive Standard	N/A	N/A	High	Unclear

Berger et al.	2017	Belgium	121	CRP/ESR/sWBC/PMN/α-Defensin/Aspiration Culture	Knee/Hip	MSIS	Yes	N/A	High	High
Rothenberg et al.	2017	USA	341	CRP/ESR/sWBC/PMN/Aspiration Culture	Knee/Hip	MSIS	N/A	N/A	High	High
Pohlig et al.	2017	Germany	20	CRP/ESR/Aspiration Culture	Hip	MSIS	N/A	N/A	High	Unclear
Fernández-Sampedro et al.	2017	Spain	495	CRP/Aspiration Culture	Knee/Hip	Compositive Standard	N/A	N/A	High	Unclear
Shahi et al.	2017	USA	195	CRP/ESR/LE/D-Dimer	Knee/Hip	MSIS	Yes	N/A	High	Unclear
Shahi et al.	2017	USA	4662	CRP/ESR/PMN	Knee/Hip	MSIS	Yes	727	High	High
Zmistowski et al.	2017	USA	129	CRP/ESR/sWBC/PMN	Knee/Hip	Intraoperative Culture	N/A	N/A	High	Unclear
Li et al.	2017	China	93	LE	Knee/Hip	MSIS	Yes	6	High	Unclear
Bonanzinga et al.	2017	Germany	156	α-Defensin	Knee/Hip	ICM	Yes	N/A	High	Unclear
Gallo et al.	2017	Czech	391	sWBC/PMN	Knee/Hip	Compositive Standard	Yes	N/A	High	Unclear
Sousa et al.	2017	Portugal	55	sWBC/PMN/sCRP	Knee/Hip	Compositive Standard	Yes	N/A	High	Unclear
Higuera et al.	2017	USA	453	sWBC/PMN	Hip	MSIS	No	0	High	Low
Koh et al.	2017	Korea	60	LE	Knee	MSIS	No	0	High	Unclear
Balato et al.	2017	Italy	51	sWBC/PMN/α-Defensin/	Knee	ICM	No	0	High	Unclear
Kawamura et al.	2017	Japan	51	sWBC/PMN/sCRP	Hip	MSIS	N/A	N/A	High	Low
Suda et al.	2017	Germany	30	α-Defensin	Knee/Hip	MSIS	N/A	N/A	High	Unclear
Wang et al.	2017	China	63	LE	Knee/Hip	MSIS	N/A	N/A	High	Unclear
Kheir et al.	2017	USA	77	LE	Knee/Hip	Intraoperative Culture	N/A	N/A	High	Low
Ruangsomboon et al.	2017	Thailand	46	LE	Knee	ICM	N/A	N/A	High	Unclear
Lausmann et al.	2017	Germany	59	LE	Knee/Hip	Compositive Standard	N/A	N/A	High	Unclear
George et al.	2017	Cleveland	44	CRP/ESR	Knee/Hip	MSIS	Yes	44	High	Unclear
Lindsay et al.	2017	USA	21	CRP/ESR	Knee/Hip	MSIS	N/A	N/A	High	Unclear

Kuo et al.	2018	China	214	CRP/ESR/sWBC/PMN/Aspiration Culture	Knee/Hip	MSIS	N/A	N/A	Unclear	Unclear
Riccio et al.	2018	Italy	73	sWBC/α-Defensin/LE/Aspiration Culture	Knee/Hip	MSIS	Yes	3	High	Unclear
De Vecchi et al.	2018	Italy	66	sWBC/sCRP/LE/α-Defensin/Aspiration Culture	Knee/Hip	ICM	Yes	N/A	High	Unclear
Fink et al.	2018	Germany	116	CRP/Aspiration Culture	Knee	Intraoperative Culture	Yes	5	High	Unclear
Fang et al.	2018	China	71	Aspiration Culture	Knee/Hip	Compositive Standard	Yes	5	High	High
Larsen et al.	2018	Denmark	96	Aspiration Culture	Knee/Hip	MSIS	N/A	N/A	High	High
Morgenstern et al.	2018	Germany	142	Aspiration Culture	Knee/Hip	EBJIS	N/A	N/A	High	Unclear
Huang et al.	2018	China	67	Aspiration Culture	Knee/Hip	Compositive Standard	N/A	N/A	High	Unclear
Renz et al.	2018	Germany	212	CRP/LE/α-Defensin	Knee/Hip	MSIS/IDSA/EBJIS	Yes	5	High	Unclear
Dwyer et al.	2018	USA	205	ESR/sWBC/PMN	Knee/Hip	MSIS	Yes	11	High	Unclear
Erdemli et al.	2018	Turkey	88	CRP/sCRP/IL-6/sIL-1β/sTNF-a/	Knee/Hip	MSIS	Yes	8	High	Unclear
Gallo et al.	2018	Czech	197	IL-6/sCRP/sIL-6	Knee/Hip	MSIS	Yes	19	High	High
Shohat et al.	2018	USA	1220	CRP/ESR/sWBC/PMN/α-Defensin	Knee/Hip	MSIS	Yes	110	High	High
Tahta et al.	2018	Turkey	38	CRP/ESR/sWBC/PMN/PCT/sCRP/α-Defensin	Knee	MSIS	Yes	38	Unclear	High
Kheir et al.	2018	USA	1095	CRP/ESR/sWBC/PMN	Knee/Hip	ICM	N/A	N/A	High	High
Kelly et al.	2018	USA	39	α-Defensin	Knee/Hip	MSIS	Yes	2	High	Unclear
Plate et al.	2018	Switzerland	109	α-Defensin	Knee/Hip	MSIS	Yes	7	High	Unclear
Stone et al.	2018	USA	183	sCRP/α-Defensin	Knee/Hip	MSIS	Yes	N/A	High	Unclear

Zahar et al.	2018	Germany	337	sWBC/PMN/LE	Knee/Hip	MSIS	Yes	N/A	High	Unclear
Balato et al.	2018	Italy	167	sWBC/PMN	Knee	ICM	No	0	High	Unclear
Sigmund et al.	2018	Germany	71	α-Defensin	Knee/Hip	MSIS/IDSA/EBJIS	N/A	N/A	High	Unclear
De Saint Vincent et al.	2018	France	41	α-Defensin	Knee/Hip	MSIS	N/A	N/A	High	Unclear
Gehrke et al.	2018	Germany	195	α-Defensin	Knee/Hip	MSIS	N/A	N/A	High	Unclear
Kanwar et al.	2018	USA	70	α-Defensin	Knee/Hip	MSIS	N/A	N/A	High	Unclear
Li et al.	2018	China	204	LE	Knee/Hip	MSIS	N/A	N/A	High	Unclear
Sebastian et al.	2018	India	40	CRP/ESR	Knee/Hip	MSIS	Yes	7	High	Unclear
Stylianakis et al.	2018	Greece	79	CRP/ESR/WBC	Knee/Hip	MSIS	Yes	2	High	Unclear
Sa-Ngasoongsong et al.	2018	Thailand	32	PCT	Knee/Hip	ICM	Yes	2	High	Unclear
Wouthuyzen-Bakker et al.	2018	Canada	52	CRP/ESR	Knee/Hip	Compositive Standard	Yes	4	High	Low
Tani et al.	2018	Greece	114	CRP/ESR	Knee/Hip	Compositive Standard	N/A	N/A	High	Low
Ettinger et al.	2019	Germany	72	α-Defensin/sIL-6/sCRP	Knee/Hip	Intraoperative Culture	N/A	N/A	High	Low
Benedetto et al.	2019	Italy	50	LE	Knee/Hip	MSIS	N/A	N/A	High	Low
Ding et al.	2019	Singapore	70	ESR/sWBC/PMN/α-Defensin	Knee/Hip	MSIS	N/A	N/A	High	Unclear
Huang et al.	2019	China	101	CRP/ESR/D-Dimer	Knee/Hip	MSIS	No	0	High	High
Kleiss et al.	2019	Germany	202	α-Defensin	Knee/Hip	MSIS	Yes	13	High	Unclear
Lazarides et al.	2019	UK	90	sWBC/PMN	Knee/Hip	MSIS	Yes	90	Unclear	Unclear
Li et al.	2019	China	439	CRP/ESR/WBC/D-Dimer/ Fibrinogen	Knee/Hip	MSIS	No	0	High	Unclear
Plate et al.	2019	Switzerland	171	sCRP	Knee/Hip	MSIS	Yes	5	Unclear	Unclear

Qin et al.	2019	China	122	CRP/ESR/D-Dimer	Knee/Hip	MSIS	No	0	Unclear	High
Salari et al.	2019	Italy	72	Calprotectin	Knee	ICM	No	0	Low	High
Schiffner et al.	2019	Germany	108	CRP/WBC	Hip	Compositive Standard	N/A	N/A	High	Unclear
Stone et al.	2019	USA	52	α-Defensin	Knee/Hip	MSIS	Yes	5	High	High
Xiong et al.	2019	China	80	CRP/ESR/D-Dimer	Knee/Hip	MSIS	No	0	Unclear	High
Xu et al.	2019	China	318	CRP/ESR/IL-6/D-Dimer/FDP	Knee/Hip	MSIS	No	0	High	Unclear
Zagra et al.	2019	Italy	119	LE	Hip	MSIS	Yes	N/A	High	Unclear
Fu et al.	2019	China	30	CRP/ESR	Knee/Hip	MSIS	No	0	High	Low
Xu et al.	2019	China	102	Fibrinogen	Hip	MSIS	N/A	N/A	Low	High
Trotter et al.	2020	UK	69	Calprotectin	Knee/Hip	ICM	N/A	N/A	High	Unclear
De Saint Vincent et al.	2020	France	106	α-Defensin	Knee/Hip	ICM	N/A	N/A	High	Unclear
Fink et al.	2020	Germany	390	CRP	Knee/Hip	ICM	No	0	High	Low
Dijkman et al.	2020	Netherlands	89	LE	Knee/Hip	MSIS	Yes	12	High	Low
Wang et al.	2020	China	63	Aspiration Culture	Knee/Hip	MSIS	N/A	N/A	High	Unclear
Bin et al.	2020	China	90	CRP/ESR/Fibrinogen	Knee/Hip	MSIS	No	0	Low	Unclear
Wu et al.	2020	China	136	CRP/ESR/Fibrinogen/D-Dimer/FDP	Knee/Hip	MSIS	N/A	N/A	High	Unclear
Xu et al.	2020	China	360	CRP/ESR/Fibrinogen/PLT	Knee/Hip	MSIS	No	0	Low	High
Bingham et al.	2020	USA	164	CRP/ESR	Knee/Hip	MSIS	No	0	Unclear	High
Qin et al.	2020	China	122	CRP/ESR/D-Dimer	Knee/Hip	MSIS	No	0	Low	High
Qin et al.	2020	China	50	CRP/ESR/PMN	Knee/Hip	MSIS	No	0	Low	High
Klim et al.	2020	Austria	124	CRP/WBC/IL-6/PCT/Fibrinogen	Knee/Hip	MSIS	No	0	High	Unclear

Paziuk et al.	2020	USA	4939	CRP/ESR/PVR	Knee/Hip	MSIS	No	0	High	Low
Pannu et al.	2020	USA	111	D-Dimer	Knee/Hip	ICM	N/A	N/A	High	High
Ackmann et al.	2020	Germany	119	CRP/D-Dimer/IL-6	Knee/Hip	MSIS	No	0	High	Unclear
Fang et al.	2020	China	38	Aspiration Culture	Knee/Hip	MSIS	No	0	Low	High
Wang et al.	2020	China	157	CRP/ESR/D-Dimer/Fibrinogen/FDP	Knee/Hip	MSIS	No	0	Unclear	High
Zhang et al.	2020	China	63	Calprotectin	Knee/Hip	MSIS	No	0	Low	High
Levent et al.	2021	Germany	260	CRP/PMN/sWBC/LE/α-Defensin	Knee/Hip	ICM	N/A	N/A	High	Unclear
Yu et al.	2021	China	139	CRP/IL-6	Knee/Hip	MSIS	N/A	N/A	High	Low
Yu et al.	2021	China	130	LE/α-Defensin	Knee/Hip	EBJIS	N/A	N/A	High	Low
Deirmengian et al.	2021	USA	228	CRP/ESR/sWBC/PMN/α-Defensin	Knee/Hip	MSIS	Yes	N/A	Low	High
Praz et al.	2021	France	152	CRP	Knee/Hip	MSIS	N/A	N/A	Low	Unclear
Grzelecki et al.	2021	Poland	133	CRP/ESR/D-Dimer	Knee/Hip	ICM	No	0	High	Unclear
Chisari et al.	2021	USA	259	CRP/ESR/ LE /Fibrinogen/ sCRP/sWBC/D-Dimer/α-Defensin	Knee/Hip	ICM	N/A	N/A	High	High
Sahin et al.	2021	Turkey	62	CRP/ESR/PVR	Knee/Hip	ICM	No	0	High	Unclear
Grzelecki et al.	2021	Poland	195	Calprotectin	Knee/Hip	ICM	Yes	25	High	Unclear
Wang et al.	2021	China	93	CRP/ESR/sIL-1β/PMN	Knee/Hip	MSIS	No	0	Unclear	High
Yin et al.	2021	China	35	CRP/ESR/IL-6/PCT/D-Dimer/ Aspiration Culture	Knee/Hip	MSIS	No	0	High	Unclear
Xu et al.	2021	China	65	CRP/ESR/IL-6/FDP/D-Dimer	Knee/Hip	ICM	Yes	N/A	High	High
Sigmund et al.	2021	Austria	176	CRP/WBC/PMN/NLR/PVR/ Fibrinogen	Knee/Hip	EBJIS	N/A	N/A	High	Low

Huang et al.	2021	China	149	CRP/ESR/PVR/Fibrinogen/D-Dimer	Knee/Hip	MSIS	Yes	64	High	Low
Qiao et al.	2021	China	210	CRP/ESR/PVR/Fibrinogen/ D-Dimer/FDP/PLT	Knee/Hip	MSIS	N/A	N/A	Low	Unclear
Wang et al.	2021	China	97	CRP/ESR/sCRP/PMN	Knee/Hip	MSIS	No	0	Unclear	High
Ivy et al.	2021	USA	99	α-Defensin	Knee/Hip	MSIS/IDSA	N/A	N/A	High	Unclear
Shohat et al.	2021	USA	122	LE/α-Defensin	Knee/Hip	ICM	N/A	N/A	High	High
Abdo et al.	2021	Brazil	53	α-Defensin	Knee	MSIS	Yes	12	High	Low
Li et al.	2021	China	50	CRP/ESR/PCT/α-Defensin/D-Dimer	Knee/Hip	MSIS	No	0	Low	High
Tirumala et al.	2021	USA	538	CRP/ESR/PMN/sWBC/PLT/PVR	Knee	ICM	N/A	N/A	High	Unclear
Warren et al.	2021	USA	123	Calprotectin	Knee	MSIS	N/A	N/A	Low	High
Ye et al.	2021	China	158	CRP/ESR/NLR	Knee/Hip	MSIS	No	0	Low	Unclear
Klemt et al.	2022	USA	464	CRP/ESR/PMN/sWBC/MLR/NLR/ PLR/PVR	Hip	MSIS	No	0	Low	High
Baker et al.	2022	USA	588	CRP/ESR/PMN/sCRP/sWBC/ α-Defensin	Knee/Hip	ICM	No	0	High	Low
Kuo et al.	2022	Taiwan, China	76	CRP/ESR/D-Dimer/sWBC/LE/ α-Defensin/PMN/Aspiration Culture	Knee/Hip	ICM	N/A	N/A	High	Low
Grassi et al.	2022	Italy	93	Calprotectin	Knee	ICM	N/A	N/A	Low	High
Shang et al.	2022	China	206	CRP/ESR/PLT/PCT/PVR	Knee/Hip	MSIS	No	0	High	Unclear
Liu et al.	2022	China	58	CRP/ESR/PCT/D-Dimer	Knee/Hip	MSIS	N/A	N/A	High	Low
Xu et al.	2022	China	543	CRP/ESR//Fibrinogen/IL-6/ PLT/MLR/NLR/PLR	Knee/Hip	ICM	N/A	N/A	High	High

Huang et al.	2022	China	99	CRP/ESR/PMN	Knee/Hip	MSIS	No	0	High	Low
Jiao et al.	2022	China	115	CRP/ESR/NLR	Knee/Hip	MSIS	No	0	Low	Unclear
Qin et al.	2022	China	70	CRP/ESR/IL-6/sIL-6/PMN/D-Dimer	Knee/Hip	MSIS	Yes	30	Unclear	High
Xu et al.	2022	China	63	CRP/ESR/Fibrinogen/NLR	Knee/Hip	ICM	N/A	N/A	High	High
Fernandez-Sampedro et al.	2022	Spain	180	CRP/ESR/D-Dimer	Knee/Hip	IDSA	No	0	Unclear	Low
Chen et al.	2022	China	186	CRP/ESR/D-Dimer/FDP/PVR/PLT	Knee/Hip	MSIS	N/A	N/A	High	Low
Chen et al.	2022	China	52	ESR/FDP/D-Dimer/Fibrinogen	Knee/Hip	MSIS	N/A	N/A	Unclear	High
Maimaiti et al.	2022	China	246	CRP/ESR/WBC/MLR/NLR/PLR/ PVR/D-Dimer/Fibrinogen/PLT	Knee/Hip	MSIS	No	0	Low	High

a. PLT: platelet count; NLR: neutrophil to lymphocyte ratio; MLR: monocyte to lymphocyte ratio; PLR: platelet to lymphocyte ratio; PVR: platelet to mean platelet volume ratio; FDP: fibrin degradation product; sWBC: synovial WBC; sCRP: synovial CRP; sIL-6: synovial IL-6; sTNF-α: synioal TNF-α; sIL-1β: synovial IL-1β; PCT: serum procalcitonin; LE: leucocyte esterase; MSIS: the Musculoskeletal Infection Society criteria; IDSA: the Infectious Diseases Society of America criteria; EBJIS: European Bone and Joint Infection Society criteria; ICM: the International Consensus Meeting (ICM); Compositive Standard: Study-defined comprehensive PJI diagnostic evaluation criteria; IA: inflammentary arthritis; QUADAS-2: The Quality Assessment of Diagnostic Accuracy Studies 2.

b. The articles report no information of the IA patients included.

c. The articles report no information of the specific number of the IA patients included.
