# Supplementary material for: Reliable Diagnostic Tests and Thresholds for Preoperative Diagnosis of Non‐Inflammatory Arthritis Periprosthetic Joint Infection: A Meta‐analysis and Systematic Review
Source: Orthop Surg. 2022 Oct 1;14(11):2822–36. doi: 10.1111/os.13500 (PMC9627080; doi:10.1111/os.13500)
Supplement: Supplementary file 3 — Appendix S3 Subgroup analysis of chronic PJI without IA population [file OS-14-2822-s004.pdf]

Appendix S3: Subgroup analysis of chronic PJI without IA population

	Studies Number	SEN (95% CI)	SPE (95% CI)	AUC (95% CI)	PLR (95% CI)	NLR (95% CI)	DOR (95% CI)	I^2 (p value)
**PMN (%)**								
60-65	5	0.89 (0.82,0.94)	0.89 (0.81,0.93)	0.95 (0.93,0.96)	7.8 (4.5,13.6)	0.12 (0.07,0.22)	64 (22,186)	0% (p=0.451)
65-70	11	0.90 (0.85,0.93)	0.84 (0.75,0.90)	0.94 (0.91,0.95)	5.7 (3.5,9.2)	0.12 (0.08,0.18)	47 (23,97)	96% (p<0.001)
70-75	10	0.89 (0.84,0.92)	0.90 (0.86,0.93)	0.95 (0.93,0.97)	9.1 (6.5,12.8)	0.12 (0.09,0.17)	74 (45,124)	80% (p=0.003)
75-80	7	0.92 (0.85,0.96)	0.81 (0.73,0.88)	0.94 (0.91,0.95)	4.9 (3.2,7.6)	0.09 (0.05,0.19)	52 (19,146)	0% (p=0.212)
80	16	0.84 (0.80,0.88)	0.94 (0.90,0.97)	0.93 (0.90,0.95)	14.6 (8.4,25.4)	0.17 (0.13,0.21)	88 (50,155)	98% (p<0.001)
**sWBC (/μL)**								
1000-2000	12	0.91 (0.83,0.95)	0.89 (0.81,0.94)	0.95 (0.91,0.97)	8.1 (4.6,14.4)	0.11 (0.06,0.19)	76 (33,175)	96% (p<0.001)
2000-3000	8	0.85 (0.81,0.89)	0.88 (0.81,0.93)	0.89 (0.86,0.93)	7.1 (4.4,11.6)	0.17 (0.13,0.21)	43 (25,74)	90% (p<0.001)
3000	19	0.88 (0.85,0.90)	0.92 (0.88,0.95)	0.94 (0.91,0.96)	11.1 (7.2,17.2)	0.13 (0.11,0.17)	83 (47,145)	97% (p<0.001)
3000-4000	6	0.90 (0.88,0.91)	0.91 (0.89,0.92)	0.96 (0.94,0.97)	9.9 (8.3,11.9)	0.11 (0.09,0.14)	89 (63,126)	89% (p=0.483)
4000-4500	2	0.91 (0.86,0.94)	0.98 (0.97,0.99)	0.96 (0.95,0.94)	50.3 (31.1,81.4)	0.09 (0.06,0.14)	540 (282,1037)	0% (p=0.498)
**CRP (mg/L)**								
<10	31	0.81 (0.77,0.85)	0.78 (0.74,0.81)	0.86 (0.83,0.89)	3.7 (3.1,4.2)	0.24 (0.19,0.30)	15 (11,21)	98% (p<0.001)
10	56	0.81 (0.77,0.85)	0.78 (0.74,0.82)	0.87 (0.83,0.89)	3.7 (3.2,4.4)	0.24 (0.20,0.29)	16 (12,20)	100% (p<0.001)
10-13	12	0.83 (0.77,0.88)	0.78 (0.70,0.84)	0.88 (0.84,0.90)	3.8 (2.7,5.2)	0.22 (0.16,0.30)	17 (10,29)	93% (p<0.001)
13-15	6	0.84 (0.78,0.89)	0.83 (0.73,0.89)	0.90 (0.87,0.92)	4.8 (3.1,7.6)	0.19 (0.13,0.27)	26 (14,46)	86% (p<0.001)
15-20	7	0.82 (0.73,0.89)	0.83 (0.79,0.86)	0.86 (0.83,0.89)	4.7 (3.7,6.0)	0.22(0.13,0.35)	22 (11,43)	44% (p=0.084)
20-25	6	0.71 (0.47,0.87)	0.69 (0.29,0.92)	0.75 (0.72,0.80)	2.2 (0.8,6.7)	0.43 (0.20,0.90)	5 (1,28)	99% (p<0.001)
25-35	2	0.86 (0.12,1.00)	0.95 (0.91,0.97)	0.96 (0.92,1.00)	17.5 (6.5,46.8)	0.15 (0.01,3.93)	117 (2,7229)	76% (p=0.008)
**ESR (mm/h)**								
20-25	7	0.85 (0.78,0.90)	0.79 (0.65,0.88)	0.89 (0.86,0.92)	4.1 (2.5,6.7)	0.19 (0.13,0.27)	22 (13,37)	90% (p<0.001)
25-30	7	0.73 (0.65,0.80)	0.82 (0.74,0.88)	0.82 (0.79,0.85)	4.2 (2.9,6.0)	0.33 (0.26,0.41)	13 (9,19)	92% (p<0.001)

30	47	0.79 (0.74,0.83)	0.78 (0.72,0.83)	0.85 (0.82,0.88)	3.5 (2.8,4.4)	0.27 (0.22,0.33)	13 (10,18)	100% (p<0.001)
30-40	15	0.77 (0.69,0.84)	0.78 (0.72,0.83)	0.84 (0.81,0.87)	3.5 (2.7,4.5)	0.29 (0.21,0.40)	12 (7,20)	96% (p<0.001)
40-50	14	0.68 (0.61,0.75)	0.79 (0.74,0.84)	0.81 (0.77,0.84)	3.3 (2.6,4.2)	0.40 (0.32,0.50)	8 (5,13)	97% (p<0.001)

IA: Inflammatory Arthritis; SEN: sensitivity; SPE: specificity; AUC: area under the ROC curve; PLR: positive likelihood ratio; NLR: negative likelihood ratio; DOR: diagnostic odds ratio; PMN%: proportion of neutrophils in synovial fluid; sWBC: synovial WBC.
