# Supplementary material for: Reliable Diagnostic Tests and Thresholds for Preoperative Diagnosis of Non‐Inflammatory Arthritis Periprosthetic Joint Infection: A Meta‐analysis and Systematic Review
Source: Orthop Surg. 2022 Oct 1;14(11):2822–36. doi: 10.1111/os.13500 (PMC9627080; doi:10.1111/os.13500)
Supplement: Supplementary file 4 — Appendix S4 Hierarchical analysis of thresholds (chronic PJI without IA population) [file OS-14-2822-s016.pdf]

Appendix S4: Hierarchical analysis of thresholds (chronic PJI without IA population)

	Studies Number	SEN (95% CI)	SPE (95% CI)	AUC (95% CI)	PLR (95% CI)	NLR (95% CI)	DOR (95% CI)	I^2 (p value)
**PMN (%)**								
<70	17	0.87 (0.82,0.91)	0.82(0.75,0.88)	0.92 (0.89,0.94)	4.9 (3.3,7.2)	0.16 (0.11,0.23)	31 (15,61)	96% (p<0.001)
72.5 (70-75)	11	0.89 (0.84,0.92)	0.90 (0.86,0.93)	0.95 (0.93,0.97)	9.1 (6.5,12.8)	0.12 (0.09,0.17)	74 (45,124)	80% (p=0.003)
≥75	25	0.87 (0.83,0.90)	0.92 (0.88,0.95)	0.94 (0.92,0.96)	10.9 (6.9,17.1)	0.14 (0.11,0.18)	76 (46,125)	99% (p<0.001)
**sWBC (/μL)**								
<4000	44	0.88 (0.84,0.90)	0.90 (0.86,0.92)	0.95 (0.92,0.96)	8.4 (6.4,11.1)	0.14 (0.11,0.18)	60 (40,91)	99% (p<0.001)
4100 (4100-4450)	2	0.91 (0.86,0.94)	0.98 (0.97,0.99)	0.96 (0.95,0.94)	50.3 (31.1,81.4)	0.09 (0.06,0.14)	540 (282,1037)	0% (p=0.498)
≥4500	3	0.60 (0.01,1.00)	0.87 (0.76,0.94)	0.88 (0.80,0.94)	4.8 (0.5,45.3)	0.45 (0.02,12.0)	11 (0,2492)	95% (p<0.001)
**CRP (mg/L)**								
≤13	96	0.82 (0.79,0.84)	0.78 (0.75,0.80)	0.87 (0.83,0.89)	3.7 (3.3,4.1)	0.23 (0.21,0.27)	16 (13,19)	100% (p<0.001)
13.5 (13-14)	3	0.88 (0.85,0.91)	0.86 (0.84,0.88)	0.94 (0.90,0.96)	6.3 (5.5,7.3)	0.14 (0.10,0.18)	47 (33,67)	0% (p=0.499)
>14	22	0.79 (0.70,0.86)	0.82 (0.72,0.89)	0.87 (0.84,0.90)	4.4 (2.7,7.1)	0.26 (0.17,0.38)	17 (8,36)	100% (p<0.001)
**ESR (mm/h)**								
<30	17	0.83 (0.78,0.87)	0.78 (0.72,0.82)	0.88 (0.84,0.90)	3.7 (3.0,4.6)	0.22 (0.17,0.28)	17 (13,22)	98% (p<0.001)
30	48	0.79 (0.74,0.83)	0.78 (0.72,0.83)	0.85 (0.82,0.88)	3.5 (2.8,4.4)	0.27 (0.22,0.33)	13 (10,18)	100% (p<0.001)
>30	30	0.74 (0.68,0.79)	0.79 (0.75,0.83)	0.84 (0.80,0.87)	3.6 (2.9,4.3)	0.33 (0.27,0.41)	11 (7,16)	99% (p<0.001)

IA: Inflammatory Arthritis; SEN: sensitivity; SPE: specificity; AUC: area under the ROC curve; PLR: positive likelihood ratio; NLR: negative likelihood ratio; DOR: diagnostic odds ratio; PMN%: proportion of neutrophils in synovial fluid; sWBC: synovial WBC.
