# Supplementary material for: Reliable Diagnostic Tests and Thresholds for Preoperative Diagnosis of Non‐Inflammatory Arthritis Periprosthetic Joint Infection: A Meta‐analysis and Systematic Review
Source: Orthop Surg. 2022 Oct 1;14(11):2822–36. doi: 10.1111/os.13500 (PMC9627080; doi:10.1111/os.13500)
Supplement: Supplementary file 5 — Appendix S5 Hierarchical analysis of thresholds with MSIS criteria as gold standard (chronic PJI without IA population) [file OS-14-2822-s008.pdf]

Appendix S5: Hierarchical analysis of thresholds with MSIS criteria as gold standard (chronic PJI without IA population)

	Studies Number	SEN (95% CI)	SPE (95% CI)	AUC (95% CI)	PLR (95% CI)	NLR (95% CI)	DOR (95% CI)	I^2 (p value)
**PMN (%)**								
<70	9	0.86 (0.82,0.90)	0.82(0.78,0.85)	0.90 (0.87,0.93)	4.8 (3.9,6.0)	0.17 (0.12,0.23)	29 (18,47)	0% (p=0.408)
72.5 (70-75)	2	0.96 (0.74,1.00)	0.87 (0.82,0.91)	0.92 (0.91,0.98)	7.5 (5.5,10.2)	0.04 (0.00,0.36)	178 (22,1418)	72% (p=0.130)
≥75	17	0.86 (0.82,0.89)	0.92 (0.86,0.95)	0.92 (0.89,0.94)	10.3 (6.1,17.4)	0.15 (0.12,0.19)	67 (37,121)	98% (p<0.001)
**sWBC (/μL)**								
<4000	25	0.86 (0.84,0.88)	0.90 (0.86,0.92)	0.92 (0.89,0.94)	8.4 (6.3,11.1)	0.15 (0.13,0.18)	55 (38,79)	97% (p<0.001)
3966	1	0.90	0.91	-	-	-	-	-
≥4500	2	0.81	0.95	-	-	-	-	
**CRP (mg/L)**								
<11	47	0.83 (0.81,0.86)	0.78 (0.75,0.81)	0.88 (0.85,0.91)	3.9 (3.4,4.4)	0.21 (0.18,0.24)	18 (15,23)	99% (p<0.001)
13.5 (11-15)	12	0.86 (0.82,0.89)	0.80 (0.71,0.87)	0.90 (0.87,0.92)	4.3 (2.9,6.4)	0.17(0.14,0.22)	25 (16,39)	96% (p<0.001)
>15	3	0.75 (0.54,0.88)	0.88 (0.10,1.00)	0.81 (0.70,0.90)	6.1 (0.2,248.0)	0.29(0.12,0.71)	21 (0,1683)	99% (p<0.001)
**ESR (mm/h)**								
<30	10	0.80 (0.71,0.87)	0.82 (0.75,0.87)	0.88 (0.85,0.90)	4.4 (3.4,5.5)	0.24 (0.17,0.35)	18 (13,24)	97% (p<0.001)
30	25	0.79 (0.72,0.84)	0.79 (0.75,0.83)	0.86 (0.82,0.89)	3.8 (3.2,4.5)	0.27 (0.20,0.34)	14 (11,19)	99% (p<0.001)
>30	17	0.75 (0.69,0.80)	0.82 (0.78,0.86)	0.86 (0.83,0.89)	4.3 (3.3,5.4)	0.31 (0.24,0.39)	14 (9,22)	96% (p<0.001)

IA: Inflammatory Arthritis; SEN: sensitivity; SPE: specificity; AUC: area under the ROC curve; PLR: positive likelihood ratio; NLR: negative likelihood ratio; DOR: diagnostic odds ratio; PMN%: proportion of neutrophils in synovial fluid; sWBC: synovial WBC.
