# Supplementary material for: Reliable Diagnostic Tests and Thresholds for Preoperative Diagnosis of Non‐Inflammatory Arthritis Periprosthetic Joint Infection: A Meta‐analysis and Systematic Review
Source: Orthop Surg. 2022 Oct 1;14(11):2822–36. doi: 10.1111/os.13500 (PMC9627080; doi:10.1111/os.13500)
Supplement: Supplementary file 6 — Fig. S1 Alpha‐defensin Laboratory test [file OS-14-2822-s001.pdf]

Supplementary Figures S1: Alpha-defensin Laboratory test

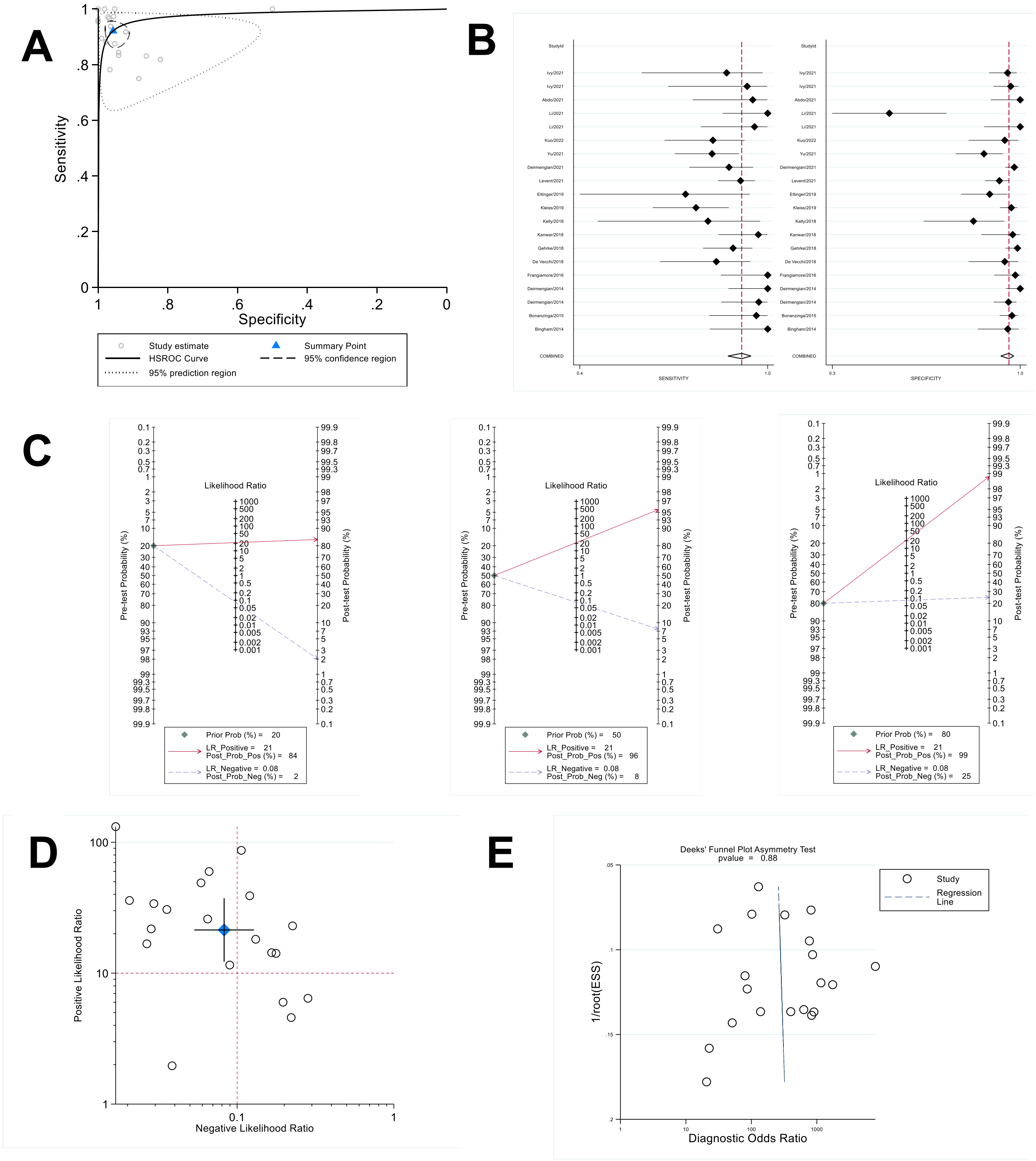

A: HSROC gram; B: Forest gram; C: Fagan's grams for low clinical suspicion of PJI (20%), low clinical suspicion of PJI (50%), and high clinical suspicion of PJI (80%); D: Likelihood Matrix gram; E: Linear regression test of funnel plot asymmetry.
